# Micro Biomimetic Eyeball for Humanoid Robots: A Visual System with High‐Density Functional Integration Based on an Origami Mechanism

**DOI:** 10.1002/advs.202515479

**Published:** 2025-10-17

**Authors:** Junji Pu, Yang Chen, Yulie Wu, Canhui Yin, Chunyan Qu, Dingbang Xiao, Xuezhong Wu

**Affiliations:** ^1^ College of Intelligence Science and Technology National University of Defense Technology Changsha 410073 China; ^2^ National Key Laboratory of Equipment State Sensing and Smart Support National University of Defense Technology Changsha 410073 China

**Keywords:** biomimetic eyeball system, bionic vision system, micro opto‐mechatronic system, origami mechanism

## Abstract

Rapid advancements in general‐purpose humanoid robots have spurred extensive in‐depth research on bionic vision systems (BVSs) with human‐like ocular functions. However, fabricating a gram‐weight miniature artificial eyeball integrated with optical imaging, dynamic field‐of‐view (FOV) modulation, and intelligent target tracking remains challenging. A biomimetic eyeball system (BES) based on the miniature origami mechanism (MOM) with three degrees of freedom (DOFs) is presented in this study to solve the inherent problems of traditional electromechanical BVSs (large volume and weight). The performance of BES in the FOV adjustment range (monocular exceeding 151.6° × 151.6°) not only precisely matches the physiological characteristics of the human eye but also offers advantages in overall size (Φ23 mm × 15 mm) and weight (1.8 g) over systems reported in current research. This system also performs high‐speed movement (saccade, 4382°/s), dynamic imaging (smooth pursuit, 532°/s), interactive control under human–robot collaboration, and active zooming. It demonstrates target recognition, locking, and tracking abilities functionally equivalent to human smooth‐pursuit movements in scenarios with active target locking based on visual attention mechanisms. This study overcomes the dual tradeoff between miniaturization and functional integrity in traditional BVSs through the novel visual solution that integrates spatial adaptability, motion compatibility, and cognitive decision‐making capabilities.

## Graphical abstract

A biomimetic eyeball system (BES) based on a 3‐DOF miniature origami mechanism, which addresses the inherent issues of large size and weight in traditional electromechanical BESs is presented. Moreover, it maintains a level of visual functionality comparable to that of the human eye, including capabilities such as rapid scanning, target recognition, and tracking.

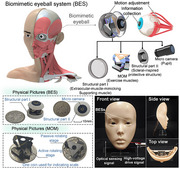

## 1 Introduction

**Figure 1:**
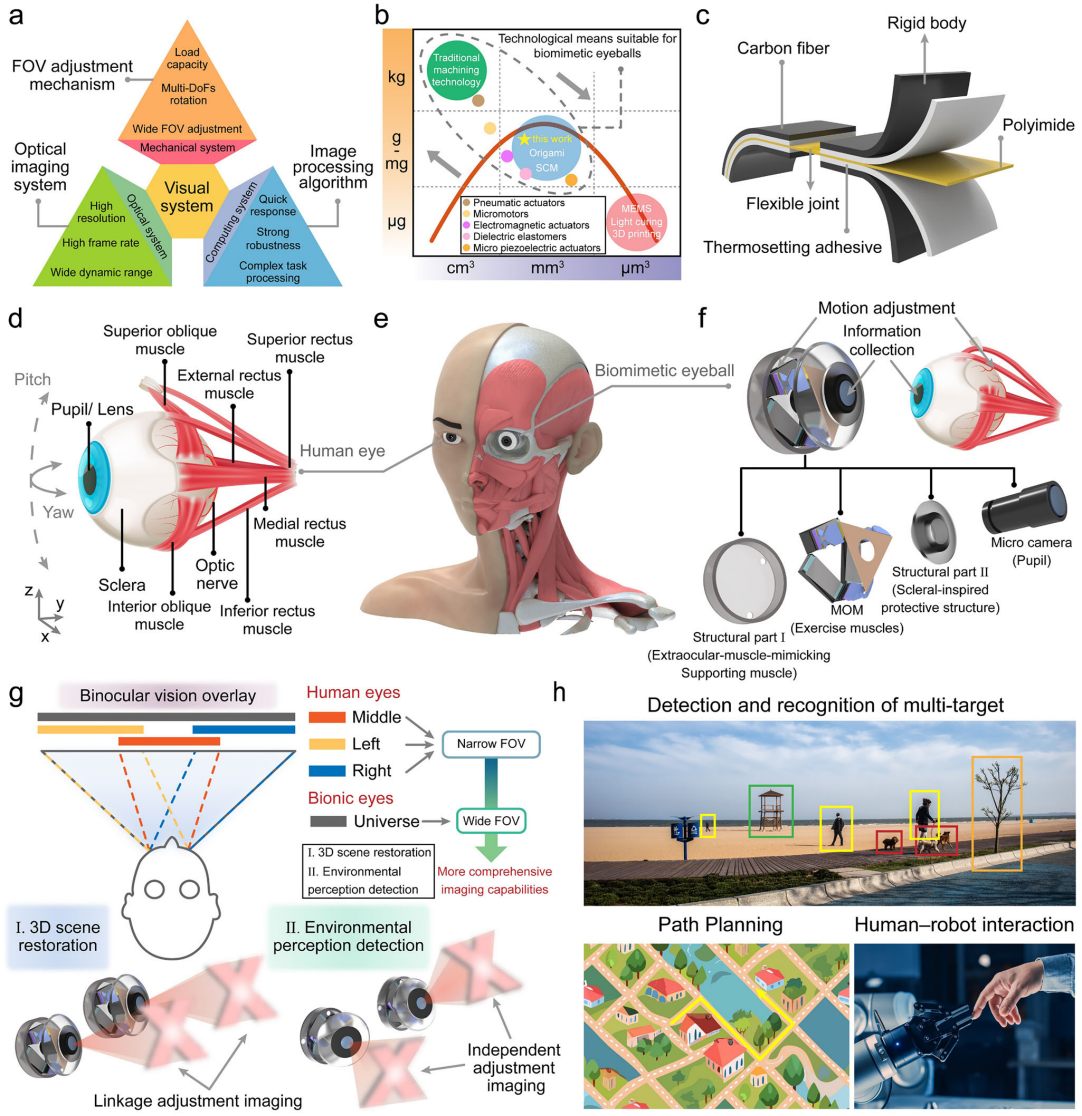
Biomimetic eyeball system (BES). a) Universal system architecture integrating an optics–mechanics–computing co‐design, which comprises an FOV adjustment mechanism, optical imaging system, and image processing algorithm. All subsystems are required to balance multiple performance characteristics. b) Technological pathway achieving gram‐scale weight and miniature dimensional co‐optimization: a novel hybrid approach combining smart composite microstructure technology/origami‐inspired multiscale fabrication and piezoelectric actuation. c) Planarized composite material processing method based on origami techniques, creating rigid‐flexible coupled microstructures through selective subtractive manufacturing. d) Anatomical features of the human eye, which include the pupil–lens imaging system, scleral protective layer, and six extraocular muscles enabling dynamic FOV adjustment. e) Size comparison between the BES and the human eye. f) BES with anatomically equivalent characteristics, integrating four core modules (microcamera, piezoelectric‐actuated MOM, sclera‐inspired support structure, and extraocular‐muscle‐mimicking support structure). g) Distinct advantages of the BVS based on biomimetic eyeballs: wide‐angle imaging capability in fully decoupled mode. h) Demonstration of extended application scenarios including multitarget detection in unstructured environments, path planning, and human–robot interaction (The pictures have been officially authorized).

Machine vision systems have demonstrated significant advantages in emerging fields such as general‐purpose humanoid robots,^[^ ^1^ ^]^ virtual reality,^[^ ^2^, ^3^ ^]^ and intelligent‐assisted driving.^[^ ^4^, ^5^ ^]^ This can be attributed to their wide‐angle adaptive adjustment, high‐speed dynamic response, and autonomous decision‐making perception capabilities, which have attracted extensive research attention. Bionic vision systems (BVSs) that emulate the wide field‐of‐view (FOV) adjustment and rapid motion tracking characteristics of biological eyes have emerged as highly promising core visual solutions for humanoid and bionic robotics.^[^ ^6^, ^7^ ^]^ However, these inherently heterogeneous, highly integrated intelligent microsystems that converge optics, mechanics, electronics, and computing suffer from formidable challenges, which include achieving collaborative optimization and deep integration of multidimensional performance metrics (**Figure** **1**a) across optical imaging systems, field of view (FOV) adjustment mechanisms, and target recognition algorithms (including lightweight compact design, high‐resolution imaging, wide‐FOV multi degrees of freedom (DOFs) dynamic regulation, and robust environmental adaptability).

The sophisticated structure and regulatory mechanisms of the human visual system offer invaluable prototypes for bionic vision technologies.^[^ ^8^, ^9^ ^]^ Anatomical studies have revealed that the human eye achieves perfect synergy between ultrawide FOV perception and millisecond‐level response speeds (Figure 1d) via its lens–retina optical imaging module, scleral support structure, and dynamic regulation system comprising six extraocular muscles.^[^ ^10^ ^]^ This naturally integrated architecture for multimodal perception and regulation provides critical inspiration for overcoming the conflict between the structural complexity and functional completeness of existing BVSs. Inspired by this, significant progress has been made in enhancing the optical imaging resolution^[^ ^11^, ^12^ ^]^ and environmental adaptability (e.g., retina‐like image sensing and biomimetic pupillary reflex systems)^[^ ^13^, ^14^, ^15^, ^16^ ^]^ and improving the robustness and computational efficiency of target recognition algorithms (e.g., lightweight neural network architectures).^[^ ^17^ ^]^ However, the high‐integration miniaturization design of FOV adjustment mechanisms remains constrained by fabrication precision and actuation paradigms of traditional moving components. Consequently, the FOV adjustment mechanisms in the current biomimetic eyeball systems (BESs) integrated into humanoid robots rely on rigid structures and micromotor actuation. Their excessive size (tens of cubic centimeters) and weight (hundreds of grams) critically constrain the available payload capacity of robot heads, precluding their application in size/weight‐sensitive platforms such as compact biomimetic robots. Recent advances in emerging manufacturing technologies such as smart composite microstructure (SCM) processing^[^ ^18^, ^19^ ^]^ and origami‐inspired multiscale fabrication^[^ ^20^, ^21^, ^22^ ^]^ have overcome limitations inherent in conventional mechanical machining, microelectromechanical systems (MEMS),^[^ ^23^ ^]^ and 3D printing technologies.^[^ ^24^ ^]^ As indicated in Figure 1b, these technologies establish a design framework for high‐precision complex mechanisms that can achieve synergistic gram‐scale weights and millimeter dimensions. This approach can be used to fabricate rigid‐flexible coupled topological structures spanning mesoscopic to microscopic scales via the selective planar removal of multilayer composites (including the rigid skeleton, flexible joint, and adhesive layers; Figure 1c). In addition, the approach provides an unprecedented co‐optimization pathway for high functional density and structural lightweighting in micromechanical systems and is the most promising solution for miniaturizing FOV adjustment mechanisms. Further, compared to pneumatic actuators,^[^ ^25^, ^26^ ^]^ micromotors,^[^ ^27^, ^28^ ^]^ electromagnetic actuators,^[^ ^29^, ^30^ ^]^ artificial muscles,^[^ ^31^, ^32^ ^]^ and dielectric elastomers,^[^ ^33^, ^34^ ^]^ miniature piezoelectric actuators^[^ ^35^, ^36^ ^]^ have emerged as core actuation solutions for micro‐origami motion structures with sub‐nanometer positioning precision, microsecond dynamic response speeds, and ultralow power‐consumption efficiency (Figure 1b).

Hence, based on biomechanical inverse design principles,^[^ ^37^ ^]^ this study established a 3D BES with anatomically equivalent features (Figure 1e). This innovative architecture includes four core modules (Figure 1f): a microcamera emulating the pupil–lens imaging function of the crystalline lens; a miniature origami mechanism (MOM) with piezoelectric actuation that minimizes actuation units to three kinematic chains through rigid‐flexible coupling topological design while maintaining precise 3 DOFs (one translational and two rotational DOFs) regulation capabilities and significantly reducing system complexity (the process of folding a 2D structure into a 3D structure can refer to Figure 7); a scleral‐inspired protective structure; and an extraocular‐muscle‐mimicking support structure.

The BES developed in this study achieved multiple key performance metrics and surpassed its biological prototype through biomimetic mapping principles. The system attains a monocular FOV of 151.6° × 151.6°, which approaches the capability of the human eye (160° × 135°) while maintaining anatomically precise dimensions (Φ23 mm × 15 mm). This topology‐optimized structure achieved significant performance advantages over existing systems, as quantified in **Table** **1**: a weight of 1.8 g and a power consumption of 275 mW at 1 Hz (Section SA, Supporting Information). For functional validation, the system demonstrated capabilities including high‐quality saccadic imaging across a wide‐angle FOV, high‐speed dynamic target imaging, interactive control under human–robot collaboration, and active zooming. The BES achieves peak angular velocities of 4382°/s (saccades, 4.8× human capacity) and 532°/s (smooth pursuit, 17.7× human capacity), significantly exceeding physiological limits. This breakthrough empowers humanoid robots with BESs to capture critical information from rapidly moving objects with unprecedented efficacy. In addition, during active target‐locking scenarios based on visual attention mechanisms, the BES demonstrates target recognition and tracking abilities that are functionally equivalent to the smooth pursuit movements in human eyes.

**Table 1:** Performance comparison of BES with other BVSs and the human eye.

Research	Drive method	Size [mm]	Weight [g]	Monocular FOV [degree]	Power [W]	Bandwidth [Hz]
BES	Piezoelectric drive	Φ23 × 15 (6.23 cm^3^)	1.8	151.6° × 151.6° (FOV) 63.6° × 63.6° (Range of motion)	Microcamera (0.264) MOM (0.011)	35.3
Human eye^[^ ^37^ ^]^	Muscle drive	SΦ25 (8.2 cm^3^)	7.5	160° × 135° (FOV) 105° × 100° (Range of motion)	≈2.5	≈5
Lotze et al.^[^ ^38^ ^]^	Motor drive	Φ40 × 40 × 40 (33.5 cm^3^, without actuating devices)	—	40° × 40° (Range of motion)	Over 45	—
Schulz et al.^[^ ^39^ ^]^	Motor drive	—(36.7 cm^3^)	258	178° × 158° (FOV) 110° × 110° (Range of motion)	Three motors (Voltage: 12–36 V and maximum current: 3 A) Camera (1.2 W)	—
Rajendran et al.^[^ ^31^, ^32^ ^]^	Artificial muscles drive	Φ40 × 200 (251.3 cm^3^)	Over 500	—	—	—
Li et al.^[^ ^34^ ^]^	Dielectric elastomers drive	763 mm^2^ × 130 (99.2 cm^3^)	—	44.8° × 44.6° (Range of motion)	Voltage: 7.2 kV	—

This system is the first successful integration of an ultracompact form with bioinspired adaptive functionalities, which helps overcome the tradeoff between miniaturization and functional integrity in conventional BVSs. This system achieves independent binocular motion‐adjustable imaging, transforming the strongly coupled narrow‐FOV imaging characteristics of human binocular vision into a fully decoupled wide‐angle imaging mode. This breakthrough demonstrates the exceptional potential of this system for 3D scene reconstruction and complex environmental perception (Figure 1g). This study also provides humanoid robots with a novel visual solution that combines spatial adaptability, motion compatibility, and cognitive decision‐making capabilities. This elevates artificial vision systems to unprecedented levels of functional integration approaching biological sensory systems, thereby demonstrating promise for deployment in more complex functional scenarios (Figure 1h).

## 2 Results

### 2.1 Design

#### 2.1.1 Design of the Miniature Origami Mechanism

**Figure 2:**
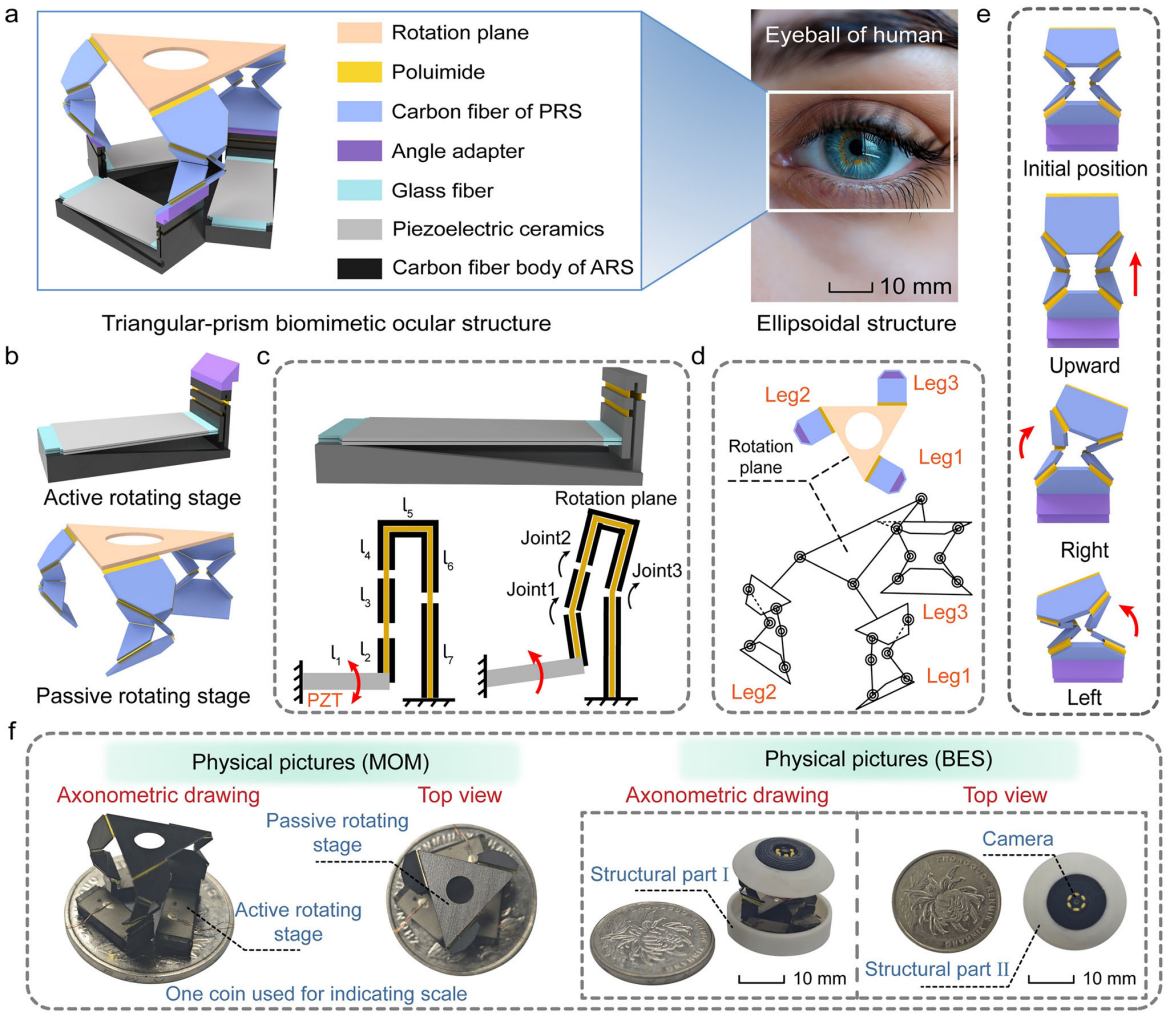
Structural characteristics and biomimetic design principles of MOM. a) Triangular‐prism origami structure emulating the ellipsoidal geometry of the human eyeball (The picture has been officially authorized). b) MOM integrates a PRS with three kinematic chains and three ARSs. c) Active four‐bar linkage rotation stage employing single‐piezoelectric actuation. d) Multi DOF kinematic chain design for the PRS, featuring seven revolute joints per chain. e) Kinematic decoupling demonstration of a single chain of the PRS, realizing independent linear and angular motion. f) Physical pictures of the MOM and BES. One coin indicates the scale.

The core breakthrough in overcoming high‐integration and lightweighting bottlenecks for BES lies in resolving the compatibility challenge of achieving large‐range dual DOFs rotation and high‐dynamic response within stringent volumetric constraints. We propose MOM, which maximizes volumetric compaction while preserving ocular‐mimetic motion functions (Φ20 mm × 12 mm, **Figure** **2**a), to accommodate the extreme spatial constraints of the ellipsoidal human eyeball. The triangular prism origami mechanism integrates three active rotating stages (ARSs) and a passive rotating stage (PRS) (Figure 2b).

The core innovation of this triangular‐prism origami mechanism lies in the co‐design of ARSs and PRS; the ARSs deliver fundamental displacement via four‐bar piezoelectric‐actuated systems (Figure 2c), whereas a PRS with three kinematic chains achieves the end displacement output (Figure 2d), with each chain containing seven revolute joints. This synergy achieves pitch, yaw, and axial motions (Movie S1, Supporting Information). Figure 2e illustrates kinematic characteristics that enable independent linear and angular motions within a single kinematic chain.

#### 2.1.2 Design of the Biomimetic Eyeball System

Based on biomechanical inverse design principles, the BES comprises 1) a microcamera for optical imaging, 2) an MOM for FOV adjustment, and 3) two skeletal structures for protection and support (Movie S2, Supporting Information). Figure 2f presents physical photographs of the MOM and BES with a dimensional reference.

### 2.2 Test and Characterization of Mechanical Properties

The enhanced mechanical properties facilitate the implementation of a simplified control strategy. To this end, a modular multiperformance testing platform is developed (Section SB, Supporting Information), and the mechanical characterization of the BES is systematically conducted. Structural optimization is achieved by quantifying the effect of hollowed dimensions of PRS kinematic chains on critical performance metrics.

#### 2.2.1 Angular Displacement and Displacement Repeatability Measurements

**Figure 3:**
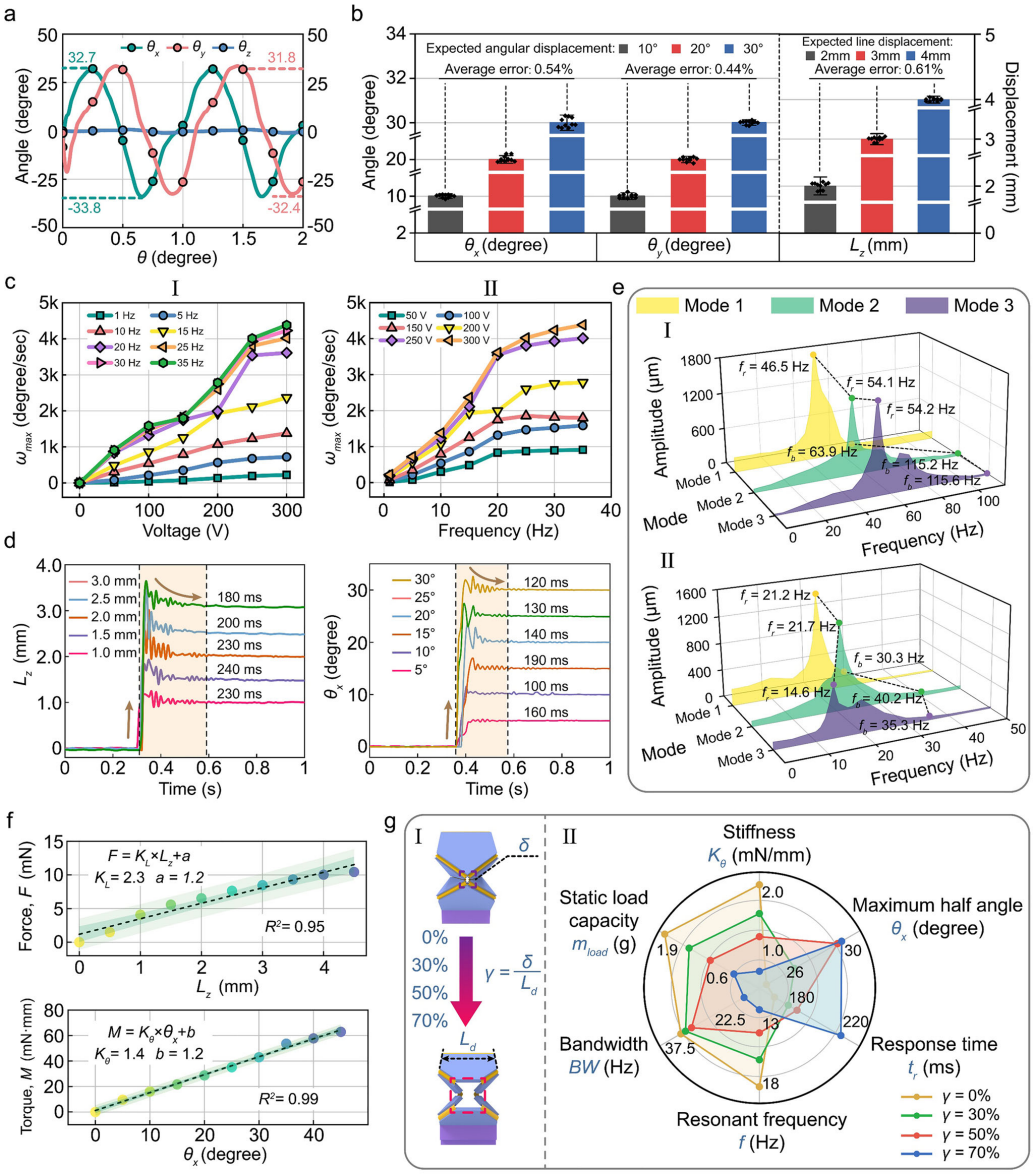
Multimodal mechanical characterization of BES. a) Dynamic response of dual‐DOF rotational angular displacement. b) Step displacement repeatability validation (intra‐group repetitions n =10). c) Peak angular velocity characteristics under drive frequency–voltage coupling (I: constant‐frequency variable‐amplitude response; II: constant‐amplitude frequency‐sweep response). d) Step responses of axial linear displacement and steering angular displacement. e) Frequency‐domain comparison between integrated/non‐integrated systems (I: MOM; II: BES). f) Axial and steering stiffness characterization via quasi‐static loading tests. g) Angular displacement optimization through geometric parameter *γ* (*γ = δ/L_d_*) (I: Topological evolution at *γ* = 0–70%; II: Comparative analysis of 6D performance).

Angular displacement testing on the BES was conducted based on the cooperative regulation mechanism of actuators. The system demonstrated exceptional dual‐DOF motion capabilities under the maximum driving voltage (300 V), achieving angular displacements exceeding ±31.8° in both pitch (*θ_x_*) and yaw (*θ_y_*) directions while maintaining roll axis (*θ_z_*) stability within 0.5° (**Figures** **3**a and 6a). Combined with the inherent 88° FOV of the microcamera, the system attained an effective visual range of 151.6° × 151.6°.

Concurrently, a stepwise open‐loop control experiment was designed by setting target displacements in the pitch/yaw (10, 20, and 30°) and axial directions (2, 3, and 4 mm) for quantifying motion accuracy. Ten repeated trials per group consistently demonstrated repeatability errors below 0.61% across all three DOFs (Figure 3b). This robust stability originated from the low motion interference rate of the system and high‐resolution actuation of the piezoelectric ceramic. Such exceptional performance substantially reduced active control complexity while enhancing the stability of the control strategy in the BES.

#### 2.2.2 Peak Angular Velocity Measurement

The BES demonstrated exceptional angular velocity regulation capabilities in the two constructed peak angular velocity test environments. Under quasi‐static conditions (*f_a_* = 1 Hz), the peak angular velocity exhibited a positive correlation with driving voltage, reaching 224°/s at a peak‐to‐peak voltage of 300 V. This characteristic extended across broader bandwidths, which suggests that flexible hinges exhibit a relatively limited hysteresis effect within a wide frequency band (Figure 3c‐I). The amplitude–frequency response analysis (Figure 3c‐II) reveals a 4382°/s peak angular velocity at 35 Hz/300 V, which is 4.8 times higher than the saccadic peak velocity of human eyes (900°/s^[^ ^40^ ^]^). This breakthrough enables rapid scene imaging and high‐speed target identification.

#### 2.2.3 Response Speed Measurement

The dynamic performance limits of BES are quantified through step response testing (Figure 3d): the axial displacement response time with a ±3% error band was 240 ± 7.2 ms, while the rotational displacement response time reached 190 ± 5.7 ms. This rotational dynamic performance surpassed the typical response range of human saccadic movements (200–250 ms).^[^ ^41^ ^]^

#### 2.2.4 Bandwidth and Stiffness Characterization

This study employed a laser displacement sensor for characterizing the frequency response of the BES under small‐displacement conditions across the three actuation modes to refine the amplitude–frequency relationship of the system (Figure 3e‐I). A control group comprising a standalone MOM without peripheral components was evaluated concurrently (Figure 3e‐II). The data indicate that pristine MOM exhibited superior high‐frequency response characteristics (operational bandwidth exceeding 63.9 Hz for all three modes). After integrating peripheral components, the bandwidth of the BES decreased to 30.3 Hz; however, it remained significantly higher than the physiological limit of the human eye (∼5 Hz).

Despite a certain degree of correlation among the system bandwidth, stiffness, and load‐bearing capacity, this study developed a quasi‐static loading‐based stiffness measurement methodology (Section SC, Supporting Information) to quantify the axial compression and steering torsion stiffness metrics. Force–displacement response curves are obtained under two characteristic working conditions of axial compression and steering torsion by integrating a uniaxial microforce sensor with a 3D precision displacement platform (Figure 3f). Experimental data indicate that BES has demonstrated exceptional linear stiffness characteristics within the workspace (compression linearity coefficient: 0.95; torsion linearity coefficient: 0.99). The precise quantification of the axial compression stiffness (2.3 ± 0.19 mN/mm) and steering torsion stiffness (1.4 ± 0.03 mN·mm/deg) provides critical parameters to optimize the design of BES.

#### 2.2.5 Optimization Strategy for the Topology Structure of the Motion Chain for Angular Displacement Performance

The angular displacement range proved decisive to achieve wide‐field imaging in the multidimensional mechanical performance of the BES. This originates from the greater practical relevance of low‐frequency wide‐angle coverage compared to that of the high‐frequency rapid saccades. Following a comprehensive characterization of the aforementioned mechanical properties, we focused on the topological configuration of the PRS, which is a critical determinant of the multi‐DOF decoupling performance. We proposed an angular displacement optimization strategy using a parametric design methodology. We established an optimization framework to enhance the angular displacement across six key mechanical performance metrics by systematically regulating the hollowed dimension ratio *γ* (γ = δ/*L_d_*; experimentally graded at 0%, 30%, 50%, 70%; see Figure 3g‐I) within the spatial folding region (*L_d_* × *L_d_*) of the kinematic chains (Figure 3g‐II).

Quantitative analysis results reveal that the system exhibits marked gradient attenuation in torsional stiffness (2.3 to 0.8 mN·mm/deg), static load capacity (2.4–0.6 g), operational bandwidth (38.5–19.6 Hz), and resonant frequency (20.2–12.2 Hz) with an increase in *γ* from 0% to 70%. The rotational angle (±24.7° to ±32.2°) and response time (172–225 ms) demonstrate upward trends. This performance shift can be attributed to the systematic structural stiffness weakening. Although expanded hollowed regions effectively mitigate stress concentration, they reduce the overall structural energy storage capacity. Under stringent volumetric constraints, the system achieves the half‐angle displacement threshold of ±30° only when *γ* ≥ 50%, with angular displacement saturating at *γ* = 50% (showing no significant enhancement beyond further hollowing). Comparative studies of polyimide films at *t_p_* = 7 and 15 µm (Section SD, Supporting Information) establish *γ* = 50% with *t_p_* = 7 µm as an optimal parameter set. All mechanical performance evaluations of the system (Figure 3a–f) are conducted based on this optimized configuration.

Furthermore, in Section SG, Supporting Information, the mechanical properties of the MOM structure were investigated through a combination of simplified theoretical analysis and finite element simulation. On one hand, the rigid flexible composite geometry, kinematics, and statics models were established for MOM; on the other hand, the influence of the parameter γ on the motion range of the system, stiffness, and operational bandwidth was quantitatively evaluated.

### 2.3 Intelligent Control and Multidimensional Functional Verification of the Biomimetic Eye System

After establishing a multimodal mechanical performance evaluation framework for BES and completing topological optimization, the viability of the biomimetic eye system as a novel robotic vision solution was validated through a tiered experimental framework. The validation addressed three pivotal scientific inquiries: i) fundamental perception and human–robot interaction capabilities under open‐loop control; ii) precision regulation performance in closed‐loop control, and iii) active target‐locking tasks based on visual attention mechanisms enabled by perception‐regulation synergy.

#### 2.3.1 Performance Evaluation of Multiscenario Open‐Loop Control

Humanoid robots executing complex interaction tasks in specific environments require their vision systems to implement a three‐level progressive perception‐control progression: establishing spatial cognition through 3D scene modeling, which enables precision physical interaction via back‐end intervention, and achieving a high‐resolution visual representation of targeted objects. This process relies on the synergistic operation of the four key open‐loop control modules: dynamic FOV scanning, which involves rapid environmental feature capture and spatial mapping; imaging critical dynamic information of a high‐speed moving target; human–robot collaborative control, which involves human intervention for effective target imaging at specific viewing angles; and active zooming, which involves observation distance dynamic adjustment to maintain clear target identification. The co‐optimization of these core functional modules determines if robots can achieve humanlike vision‐action closed‐loop functionality in unstructured environments.

##### Dynamic Field of View Scanning Test

**Figure 4:**
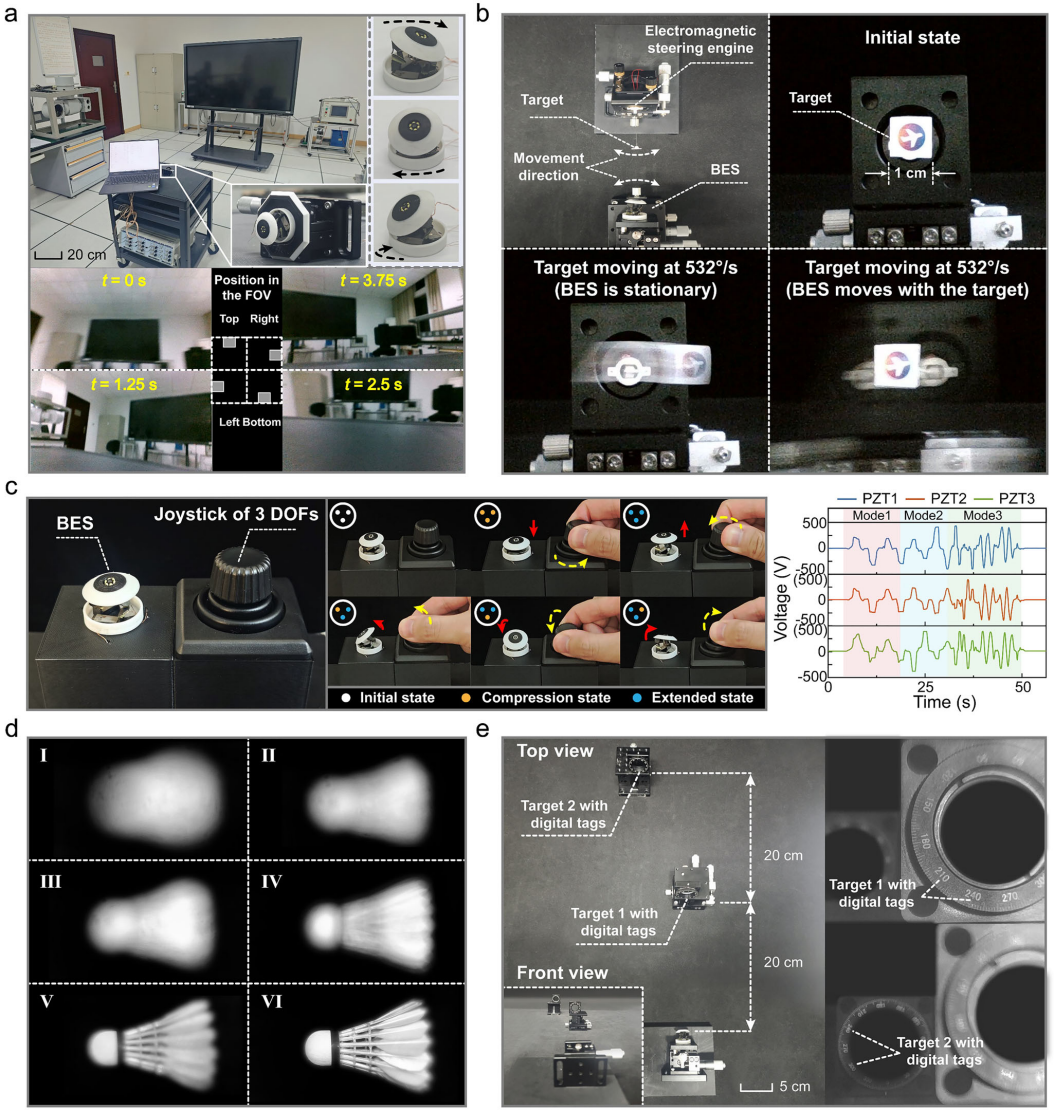
Verification of open‐loop control performance in multiple scenarios. a) Wide‐FOV dynamic scanning imaging. b) Imaging of a high‐speed moving target (532°/s). c) Real‐time interactive manipulation under human–robot collaboration. d) Single‐target active zoom verification (I–VI: Zoom process from blurred to clear). e) Active zoom verification for targets at varying distances (focal adjustment for the clear imaging of objects with labels at different distances).

We established a vision verification platform based on multiple target scenarios for evaluating the dynamic FOV imaging capabilities of the BES (**Figure** **4**a; Movie S3, Supporting Information). The system accomplished a continuous coverage of a 151.6° × 151.6° wide‐angle FOV at a 0.2 Hz scanning frequency by regulating bionic motion patterns (horizontal/vertical single‐DOF and circumferential dual‐DOF modes). The results demonstrated that this wide‐FOV imaging capability ensured complex scene reconstruction, thereby enabling the subsequent validation of other optical perception functionalities.

##### Rapid Imaging of the High‐Speed Moving Target

Capturing critical dynamic information from rapidly moving objects is essential, in addition to the high‐fidelity static scene reconstruction. We assess the BES performance in this regime using a target affixed to a high‐speed oscillating electromagnetic steering engine (Figure 4b). Under the stationary BES operation, a target traversing at 532°/s generated pronounced motion blur, obscuring its identifiable features. In contrast, the system achieved a clear imaging of the moving target when the BES driving frequency and phase were actively synchronized with the motion profile of the target (Movie S4, Supporting Information). The motion compensation rendered the target effectively stationary relative to the center of the view while inducing a relative motion blur in a static background. This demonstrated the capability for high‐speed dynamic imaging surpassing the inherent physiological tracking limits of the human eye (17.7× the human eye^[^ ^42^ ^]^), and this can help humanoid robots accurately capture critical information from fast‐moving objects.

##### Real‐Time Human–Machine Interaction Control Verification

Despite the high autonomy and cost‐effectiveness demonstrated by fully autonomous robots in domains such as smart homes^[^ ^43^, ^44^ ^]^ and agricultural irrigation,^[^ ^45^, ^46^ ^]^ robotic vision systems operating in unstructured environments such as disaster rescue^[^ ^47^ ^]^ and deep‐sea exploration^[^ ^48^, ^49^ ^]^ require the synergistic integration of autonomous decision making and human intervention. To address this issue, we established a human–robot collaborative verification framework for the BES (Movie S5, Supporting Information). The independent precision control of three actuators was achieved by developing a collaborative control interface centered on a 3‐DOF mechanical joystick and leveraging a hardware‐in‐the‐loop simulation platform for signal modulation and amplification. Figure 4c shows seamless transitions across the three operational modes, which display driving voltage inputs for all actuators and kinematic chain states (initial, compressed, and extended) of the PRS. This collaborative capability provides essential methodological support for the targeted imaging of specific views.

##### Active Focusing Test

We validated the human‐eye‐like autofocus capability of the BES after building up the open‐loop control capabilities for wide FOV coverage and specific perspective adjustment. The optical lens was integrated into the upper plane of MOM by utilizing a modular split architecture, whereas the image sensor remained fixed on the base directly beneath it. The precise regulation of the lens‐sensor spacing (*L_z_*) achieved clear imaging for a single target and multiple objects at different distances by leveraging single‐DOF axial motion characteristics of the MOM (Figure 4d,e). This helped verify the inherent advantages of the BES in adaptive optical autofocusing.

#### 2.3.2 Performance Characterization of Closed‐Loop Feedback Control

**Figure 5:**
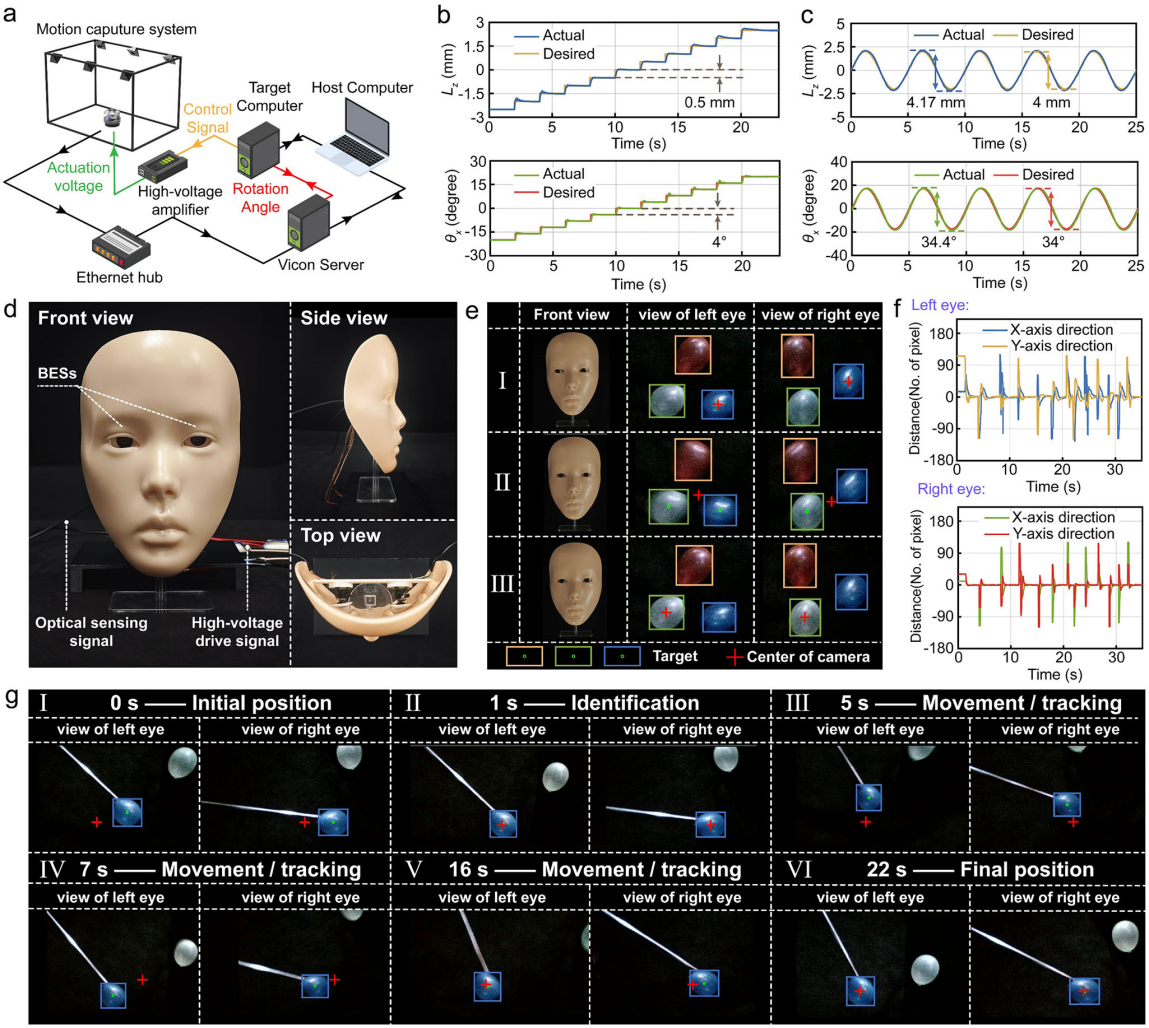
Closed‐loop feedback control performance validation of BES. a) Architecture of closed‐loop experimental platform (incorporating a multicamera motion capture system: OptiTrack PrimeX 41). b) Step response characteristics for axial linear displacement (±2.5 mm, step size: 0.5 mm) and pitch angular displacement (±20°, step size: 4°). c) Displacement tracking performance (Input signal frequency: 0.2 Hz, phase delay less than 90 ms). d) Bionic facial platform integrated with a pair of BESs. e) Multitarget recognition and locking based on visual attention mechanisms. f) Visual deviation during active target locking (Offset between the view center of cameras and target centers quantified by pixel distance). g) Single‐target tracking verification via visual attention mechanisms (Test object: moving balloon).

A closed‐loop control verification framework for a BES was established by building on the aforementioned open‐loop control functionalities (**Figure** **5**a). The system achieved submillimeter motion capture accuracy for a BES equipped with three retroreflective markers through the synergistic operation of a six‐camera motion capture system (OptiTrack‐PrimeX 41) and professional analysis software (Motive). Real‐time controllers implement closed‐loop control logic through the synchronous processing of sensor data and actuator signals. Applying 0.5 mm and 4° step signals to axial (±2.5 mm) and pitch (±20°) DOFs demonstrated precise linear and angular displacement regulations, respectively (Figure 5b). Further validation with 0.2 Hz desired displacement signals (Figure 5c) revealed amplitude errors below 4.3% (linear) and 1.2% (angular), with phase delays under 90 ms (axial: 86 ms and steering: 84 ms).

#### 2.3.3 Active Target Locking Based on a Visual‐Attention Mechanism

For BVSs, the core challenge lies in converting optical information into environmentally cognizant representations with decision‐making values, which subsequently enables complex real‐world interactive tasks such as facial expression recognition,^[^ ^50^, ^51^ ^]^ multicategory object identification,^[^ ^52^, ^53^ ^]^ and visual attention following.^[^ ^54^ ^]^ To address this issue, we employed an integrated bionic facial platform (Figure 5d) to simulate humanoid–robot visual environments that validate the active target‐locking functionality of the system. The experiments utilized tricolor targets (red, blue, and green balloons) for constructing static scenes that achieve the autonomous switching of the FOV centers of dual bionic eyeballs through external command triggering. The system accurately identified and locked designated targets by leveraging the visual attention mechanism based on target recognition algorithms (Figure 5e; Movie S6, Supporting Information). Figure 5f quantifies pixel‐distance offsets between the view centers of microcameras and recognized target centers during active target switching across both BESs.

#### 2.3.4 Trajectory Tracking of a Single Target

Building upon the established active target‐locking mechanism, this section equips the BES with human eye‐like visual tracking capabilities. For the experimental validation of the continuous tracking performance, a dynamic target (blue balloon) was employed within a motion scenario. Real‐time state transitions of the system were recorded across feature recognition, target locking, and continuous tracking stages using a three‐stage progressive verification method (Positions I–VI) (Figure 5g; Movie S7, Supporting Information). The results demonstrate that the system can autonomously perform full‐process tracking tasks, replicating the biological functionality of human saccadic smooth‐pursuit movements.

## 3 Discussion

During long‐term operation of the BES, large‐angle cyclic folding deformation of the flexible hinge may lead to adhesion failure at the interface between rigid and flexible materials, which represents a critical factor affecting the service life of the system. To enhance interfacial durability, this study proposes the use of adhesion promoters to improve the interlaminar toughness of the composite material. It is noteworthy that for humanoid robots operating in complex environments, long‐term system stability, including the fatigue life of the flexible hinge and overall reliability, is particularly important. Preliminary test data (Section SE, Supporting Information) indicate that the basic structural unit can withstand over 36 000 cycles at a bending angle of 150° without significant performance degradation, and the full system maintains stable motion output after continuous operation for 3 h.

On the other hand, external vibration represents a common technical challenge (Section SF, Supporting Information) for rigid‐flexible composite micro‐mechanical systems. Based on the existing PZT actuation framework, the vibration suppression strategy that combines enhanced passive damping characteristics with active feedback control may be an available method. Specific solutions include optimizing joint dynamic performance using a “flexible‐damping‐flexible” triple‐layer composite structure, as well as implementing closed‐loop attitude control via embedded angle sensors.

In future work, by integrating multi‐source sensory information such as visual data, inertial measurement unit (IMU) data, and joint angle feedback, the environmental adaptability and control precision of the BES are expected to be significantly improved. This multi‐modal sensing architecture offers a feasible technical pathway for advanced tasks in next‐generation humanoid robots, such as scene understanding and autonomous navigation.

## 4 Conclusion

Inspired by human ocular characteristics, this study employed a bionic topology‐driven co‐design strategy and developed a novel ocular architecture anatomically equivalent to the human eye. The functional integrity of this system as a humanoid robot visual scheme was fully validated after integrating multiple components, including a miniature FOV adjustment mechanism, microcamera, and sclera‐inspired and extraocular muscle‐inspired support structures.

To overcome the volumetric and power consumption limitations of conventional BVSs, this study innovatively integrated piezoelectric actuation with origami fabrication for developing a 3‐DOF MOM. Utilizing a modular testing platform, we characterized multiple mechanical properties of this system, which include angular displacement and response speed. Through the development of a performance evaluation framework based on six fundamental mechanical metrics, the critical angular displacement was enhanced, leading to the topological optimization of key kinematic chains. The results demonstrate that BES owns significant advantages compared to those in current research in total size (Φ23 mm × 15 mm), weight (1.8 g), and power consumption (1 Hz, 275 mW), while achieving compound pitch‐yaw motion exceeding ±31.8°. The scanning peak angular velocity capacity of the BES (4382°/s, 4.8× human capacity) provided the foundation for imaging high‐speed dynamic targets.

Building on the superior mechanical performance of the BES, we demonstrated its application potential in intelligent control and multidimensional scenarios, thereby validating its functional integrity. Multimodal open‐loop control assessments revealed its unique advantages in high‐quality saccadic imaging across wide fields (monocular 151.6° × 151.6°), high‐speed dynamic target imaging, human–robot collaborative interaction, and active zooming (±5.3 mm), which are capabilities essential for target‐specific imaging in collaborative scenarios. The high‐speed dynamic imaging capability of the BES (532°/s, smooth pursuit) exceeded the physiological limits of the human eye by 17.7 fold, which enables humanoid robots with BESs to accurately capture critical information from fast‐moving objects. In addition, it accomplished rapid multitarget identification, active locking, and trajectory tracking by leveraging visual attention‐based target recognition algorithms, replicating smooth human pursuit movements. This foundation can enable future deployment in complex scenarios, including facial expression recognition and visual navigation.

This study provides humanoid robots with a novel visual solution that integrates spatial adaptability, motion compatibility, and cognitive decision making to achieve unprecedented functional integration approaching biological sensory sophistication. In addition to the potential research directions expressed in the discussion section, future research is also expected to focus on two primary directions: integrating bionic binocular vision algorithms to achieve ultraprecise 3D scene reconstruction and exploring enhanced architectures that enable multidistance focusing while maintaining wide‐FOV imaging capabilities. These explorations will not only propel BVSs beyond functional imitation toward high‐performance realization but also unlock new dimensions for spatially adaptive vision technologies.

## 5 Experimental Section

### Cooperative Regulation Mechanism of Actuators to Realize Multiple Motion Modes of Biomimetic Eyeball System

Mature commercial gimbals enable rapid FOV orientation switching through input‐signal modulation. However, the BES faces stricter dynamic constraints. Although the full amplitude‐phase modulation of three‐channel drive signals could achieve precise visual field regulation, it imposes extreme demands on the dynamic modeling of miniature rigid‐flexible coupled mechanisms.

**Figure 6:**
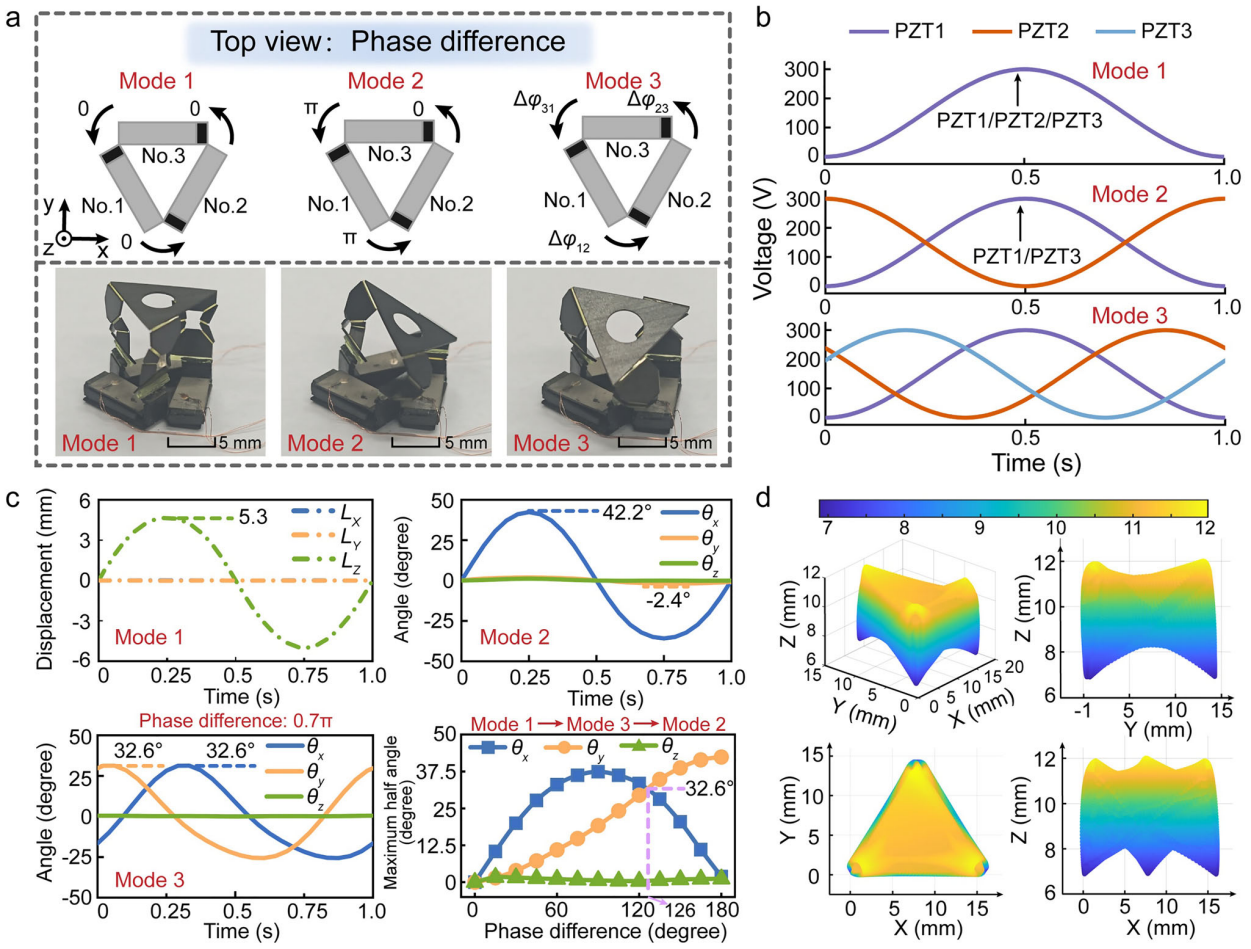
Dynamic phase‐synergy regulation mechanism of MOM. a) Phase‐difference control strategy for multimodal actuation; b) Input voltage waveforms across multiple modes (exemplified by sinusoidal signals); c) Numerical simulation of multimodal displacements: ±5.3 mm lineal travel for single‐DOF axial translation mode, ±42.2° angular travel for single‐DOF rotation mode, ±32.6° dual‐axis angular travel for dual‐DOF rotation mode (Δ*φ* = 0.7π). Phase difference (Δ*φ*) adjustment enables inter‐modal switching; d) 3D workspace of the miniature origami mechanism in dual‐DOF rotation mode (Δ*φ* = 0.7π).

To this end, a phase‐synergy simplification strategy based on parallel kinematics was proposed. The rapid switching of multiple motion modes was achieved by controlling dynamic phase differences (Δ*φ*) between actuation units under fixed drive amplitudes (**Figure** **6**a). This approach significantly reduced control complexity within acceptable error margins (coupling ratio of concomitant/effective angular displacement of less than 5.8% per DOF). The phase‐difference motion mode mapping was established as follows:

- Axial translation mode (Mode 1: Δ*φ_12_* = Δ*φ_13_* = Δ*φ_23_* = 0°). Producing vertical axial displacement (±5.3 mm stroke) for micro‐FOV focal correction.
- Single‐DOF rotation mode (Mode 2: Δ*φ_23_* = 0, Δ*φ_12_* = Δ*φ_13_* = π). Generating pitch or yaw motion about transverse axes (±42.2° angular travel) for single‐FOV scanning.
- Dual‐DOF rotation mode (Mode 3: Phase differences distinct from those of modes 1 and 2). Enabling pitch–yaw compound motion (±32.6° maximum angular travel per axis) for wide‐area environmental perception.

Figure 6b shows the input voltage waveforms that correspond to each operational mode. Constrained by the safety threshold of the PZT‐5H piezoelectric ceramics, sinusoidal drive signals peaking at 300 V maximize the angular displacement output, which helps establish a clear physical boundary for the operational limits of the BES. Although Mode 3 could be achieved when the phase differences of the three actuators are different from those of Modes 1 and 2, this study adopts a fixed phase difference method for achieving good displacement periodicity, i.e., Δ*φ_12_* = Δ*φ_13_* = Δ*φ_23_*. It is not difficult to observe from the simulation results (Figure 6c) that there are accompanying angular displacements *θ_y_* and *θ_z_* in addition to the expected effective angular displacement *θ_x_* when the system is in Mode 2. This could be attributed to the insufficient kinematic decoupling through phase‐only modulation, which required additional amplitude regulation. However, a low coupling ratio of 5.8% remained practically tolerable. Further, adjusting the fixed phase differences in Mode 3 enabled transitions from Modes 1 to 3 and then to Mode 2. At Δ*φ* = 0.7π (where Δ*φ* = Δ*φ_12_* = Δ*φ_13_* = Δ*φ_23_*), Mode 3 achieved a balanced dual‐axis angular displacement (±32.6° per axis). Synchronized workspace simulations were conducted for the miniature origami mechanism in dual‐DOF rotation mode (Δ*φ* = 0.7π, angular travel ±32.6° × ±32.6°, Figure 6d) to evaluate 3D imaging perspectives.

### Manufacturing the Biomimetic Eyeball System

**Figure 7:**
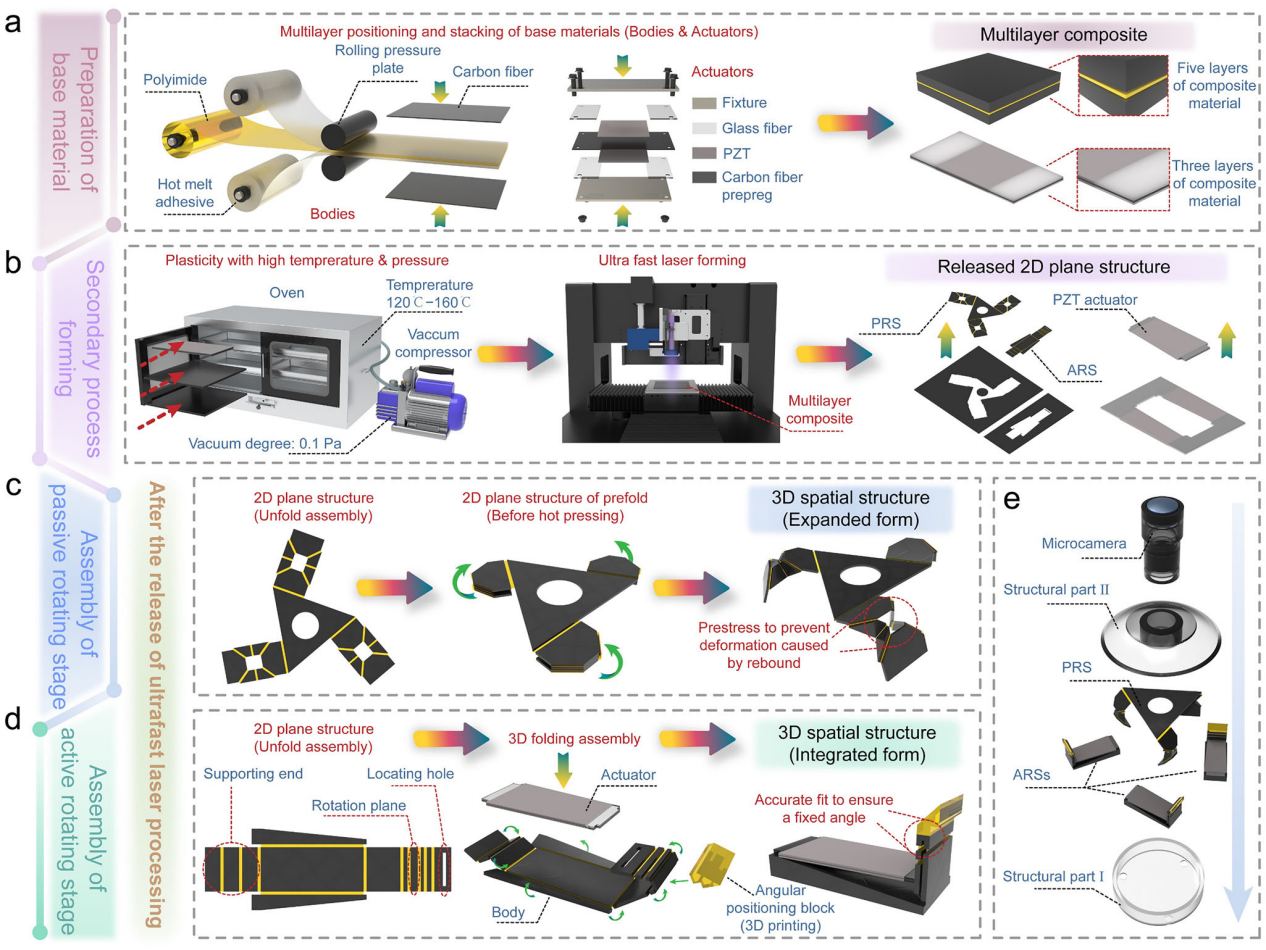
Manufacturing of BES. a) Laminate fabrication of multilayer composites: Fuselage skeleton (carbon fiber‐hot melt adhesive‐polyimide‐hot melt adhesive‐carbon fiber) and driver substrate (PZT/glass fiber‐carbon fiber prepreg‐PZT/glass fiber); b) Synergistic high‐temperature and high‐pressure forming and femtosecond laser precision machining for 2D pre‐assembled structures; c) Prestress‐induced forming process for PRS; d) 3D folding of ARSs with 3D‐printed angular positioning blocks; e) Multimodule collaborative assembly.

The key manufacturing processes for BES are illustrated in **Figure** **7**. The three core stages of the manufacturing process include i) precise fabrication of composite substrate materials, ii) functional structural formation, and iii) system integration and assembly. Two functionally graded composite systems were developed during substrate preparation (Figure 7a).

System 1: Polyimide film/carbon fiber‐reinforced layered architecture (total thickness is ≈300 µm) integrating outer carbon fiber layers (two plies), adhesive bonding layers (two plies of high‐temperature hot‐melt adhesive), and a central polyimide film layer (single‐ply), which helps create a high‐modulus fuselage skeleton enabling secondary flexible development.
System 2: Heterogeneous integration of piezoelectric ceramic (PZT‐5H)/glass fiber/carbon fiber prepregs for constructing electromechanically coupled driver substrates. The coplanar alignment between the PZT plates and glass fibers is achieved via custom fixtures.

The composite forming process employs a high‐temperature and high‐pressure co‐forming strategy (Figure 7b) for achieving a dense interlayer bonding of heterogeneous materials under a 120–160 °C thermal field and 0.1 Pa high‐vacuum environment. Subsequently, ultrafast femtosecond laser processing at a wavelength of 347 nm is used for fabricating 2D pre‐assembled structures, including ARSs, PRSs, and piezoelectric actuators, which help ensure submicron precision. As shown in Figure 7c, prestress loading is applied to the PRS to effectively suppress reverse buckling in kinematic chains during rotational bending. This involves pre‐folding rigid links along flexible joints (analogous to origami creases), followed by secondary high‐temperature hot pressing to induce an initial stress in the joints. Subsequently, 3D‐printed angular positioning blocks establish an initial angular difference (*θ_ori_* = 45°) between ARSs and PRS, which helps enable the full‐stroke utilization of piezoelectric actuator displacement (Figure 7d).

As shown in Figure 7e, the final integration employs a machine vision‐based precision assembly system to achieve the submillimeter spatial matching of the microcamera (Φ 5 mm), protective structure, ARSs, PRS, and support structure, thus completing the functional integration of BES.

## Conflict of Interest

The authors declare no competing interests.

## Author Contributions

Y.W., D.X., and X.W. conceived the study. J.P. and Y.C. performed the experiments and data measurements, analyzed the data, and wrote the manuscript. C.Y., C.Q., Y.W., D.X., and X.W. provided valuable suggestions for the experiments. All authors discussed the results and contributed to the manuscript.

## Supporting information



Supporting Information

Supplemental Movie 1

Supplemental Movie 2

Supplemental Movie 3

Supplemental Movie 4

Supplemental Movie 5

Supplemental Movie 6

Supplemental Movie 7

## Data Availability

The data that support the findings of this study are available from the corresponding author upon reasonable request.
