# Supplementary material for: Micro Biomimetic Eyeball for Humanoid Robots: A Visual System with High‐Density Functional Integration Based on an Origami Mechanism
Source: Adv Sci (Weinh). 2025 Oct 17;13(1):e15479. doi: 10.1002/advs.202515479 (PMC12767056; doi:10.1002/advs.202515479)
Supplement: Supplementary file 1 — Supporting Information [file ADVS-13-e15479-s007.docx]

**SUPPLEMENTARY INFORMATION**

**Title:**

The micro biomimetic eyeball for humanoid robots: A visual system with high-density functional integration based on an origami-mechanism

**Authors:**

Junji Pu^1,2^, Yang Chen^1,2^, Yulie Wu^1,2^*, Canhui Yin^1,2^, Chunyan Qu^1,2^, Dingbang Xiao^1,2^*, Xuezhong Wu^1.2^

First author (email: pujunji21@nudt.edu.cn)

*Corresponding author (email: [yuliewu_nudt@163.com](mailto:yuliewu_nudt@163.com); dingbangxiao@nudt.edu.cn)

**Affiliations:**

^1^College of Intelligence Science and Technology, National University of Defense Technology, Changsha 410073, China

^2^National Key Laboratory of Equipment State Sensing and Smart Support, National University of Defense Technology, Changsha 410073, China

**Supplementary Notes**

**Section SA:**

**Power characterization of the biomimetic eyeball system (BES)**

The core advantage of piezoelectric actuation lies in its inherent low-power characteristics. To quantify the maximum energy consumption of the system, we conducted power measurements for one piezoelectric actuator and the integrated BES using a high-precision power analyzer, as shown in **Figure S1a**. Experimental data reveal a quasi-linear increase in peak power consumption of single actuator from 3.67mW to 63.91mW when driving frequency escalates from 1Hz to 100Hz. Building upon single-actuator results, synchronous measurements were performed on the complete system incorporating three piezoelectric actuators and imaging modules. Remarkably, the total power consumption at 1Hz operating frequency measures merely 275mW—significantly lower than the basal metabolic consumption of single human eye. It also demonstrates technical advantages over recently reported BESs.


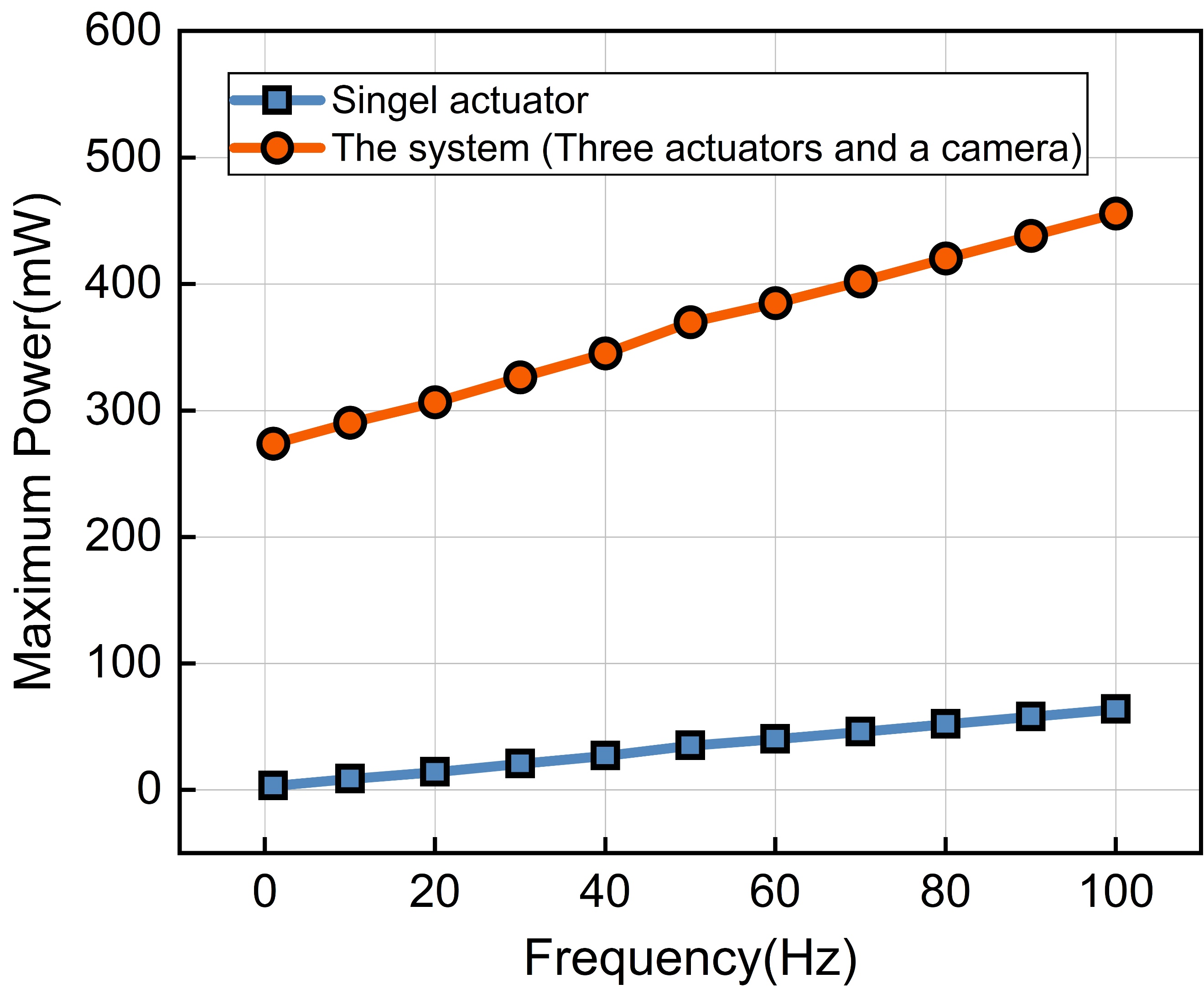


**Figure S1. Test and characterization of the system power.**

**Section SB:**

**Modular multi performance testing platform**

Comprehensive performance evaluation of MOM and BES involves measuring multiple kinematic (angular displacement, response speed, etc.) and mechanical (stiffness, load capacity, etc.) properties. Consequently, a plug-and-play modular multi-functional testing platform was developed for rapid multi-performance characterization, as detailed in **Figure S2a**.

Centered on a high-precision triaxial positioning system, this platform integrates rapidly interchangeable measurement modules: the laser displacement sensor (Shenzhen Sincevision Technology-SGI055; 0.1 μm resolution, 5 kHz bandwidth) or the micro-force sensor (Futek-LSB200; 0.1N capacity) coupled via standardized interfaces to cantilever adjustment brackets. This configuration enables seamless switching between submicron positioning and millinewton force measurements. During testing, the specimen positioned by a uniaxial lift stage (Daheng Optics) receives excitation signals from a three-channel piezoelectric drive system (CoreMorrow-EOC.C12), facilitating simultaneously acquisition of kinematic (angular displacement, saccadic velocity) and mechanical (stiffness, load capacity) parameters. **Figure S2b** illustrates the non-contact angular displacement detection scheme using the laser displacement sensor.


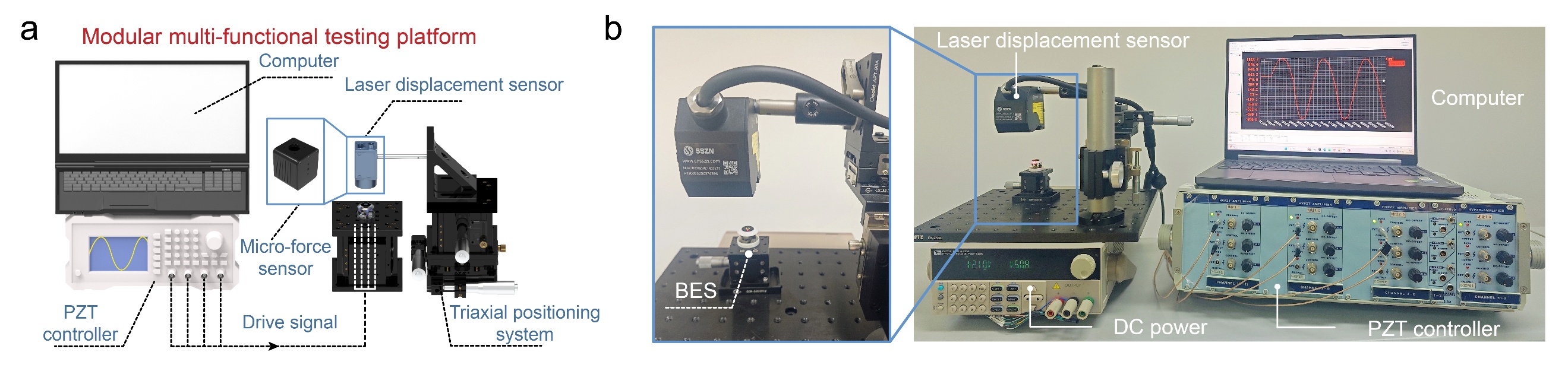


**Figure S2. Modular multi-performance test platform. a,** The construction principle of the test platform；**b,** Non-contact angular displacement detection scheme based on the laser displacement sensor.

**Section SC:**

**Stiffness measurement method based on quasi-static loading principle**

Taking stiffness testing of BES as an example, the quasi-static loading-based stiffness measurement method illustrated in **Figure S3** is implemented.

In the initial state, BES is fixed on a horizontal base (**Figure S3a**). To eliminate external vibration interference, the base may be mounted on an air-floating platform. For axial compression stiffness testing, the micro-force sensor is positioned directly above the geometric center of BES via a high-precision triaxial positioning system. Through quasi-static loading, the compression height (*L_z_*) is progressively increased while recording feedback force outputs at multiple compression heights, enabling precise evaluation of axial stiffness (**Figure S3b**).

For steering torsion stiffness characterization, an extended torsion bar ensures continuous contact between the micro-force sensor and the system. During the vertical descent of the torsion bar, transverse position adjustments via the triaxial positioning system maintain alignment between the bar tip and the sensitive surface of the sensor. Feedback moment outputs at varying compression angles *θ_x_* (*θ_x_* = *a*sin(*L_z_* / *W*)) are then acquired for accurate torsional stiffness assessment (**Figure S3c**).


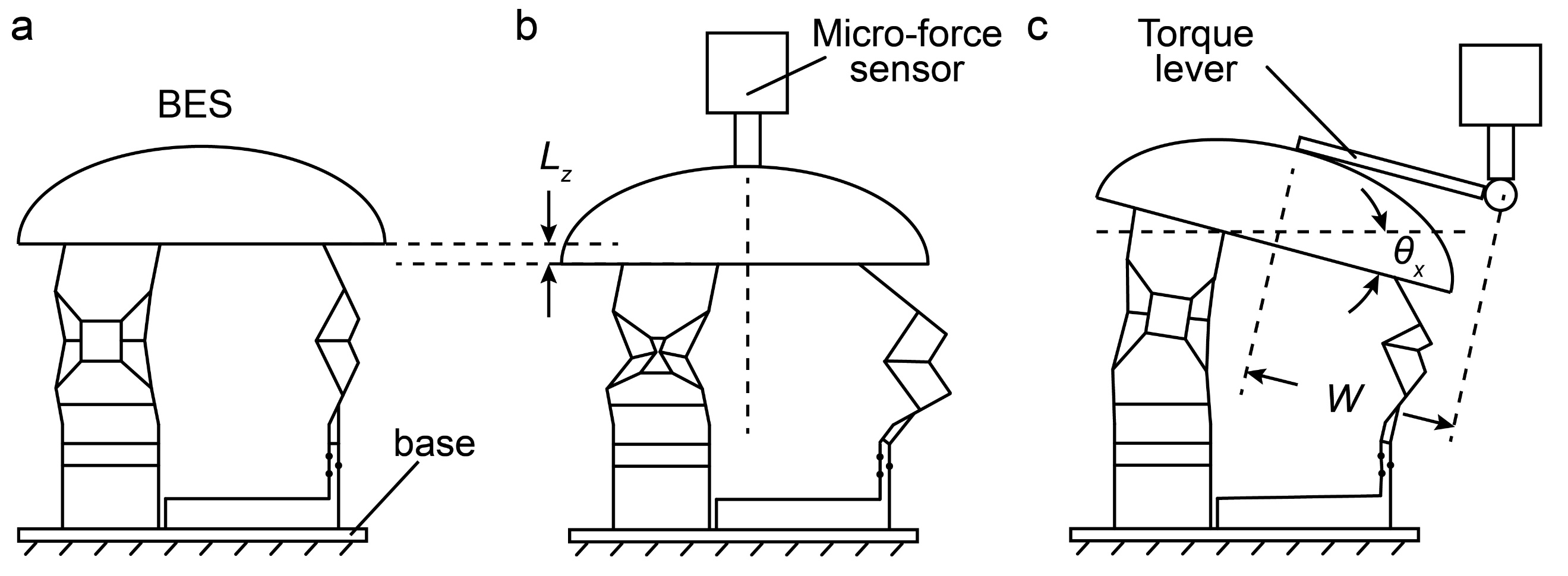


**Figure S3.** **Stiffness measurement method based on the principle of quasi-static loading. a,** Tested BES in the initial position; **b,** Axial compression stiffness measurement; **c,** Steering torsion stiffness characterization.

**Section SD:**

**Coupling optimization of motion chain topology-membrane thickness parameters**

While mechanical performance testing in the main text optimized kinematic chain hollow structures through six key metrics, this section further reveals the energy-matching criterion for flexible film thickness, which is an often-overlooked core parameter. Theoretical analysis indicates that elastic potential energy stored/released during film deformation must dynamically balance input energy from piezoelectric actuators to enhance system energy transfer efficiency. Among the geometric dimensions, film thickness proves mostly critical for elastic potential energy. Hence, we conducted multidimensional optimization for *t_p_* =7 μm and *t_p_* =15 μm (the thickness exceeding 15 μm will seriously affect the output angular displacement, so that, no comparison of thicker film sizes is made in this section), as detailed in **Figure S4**.


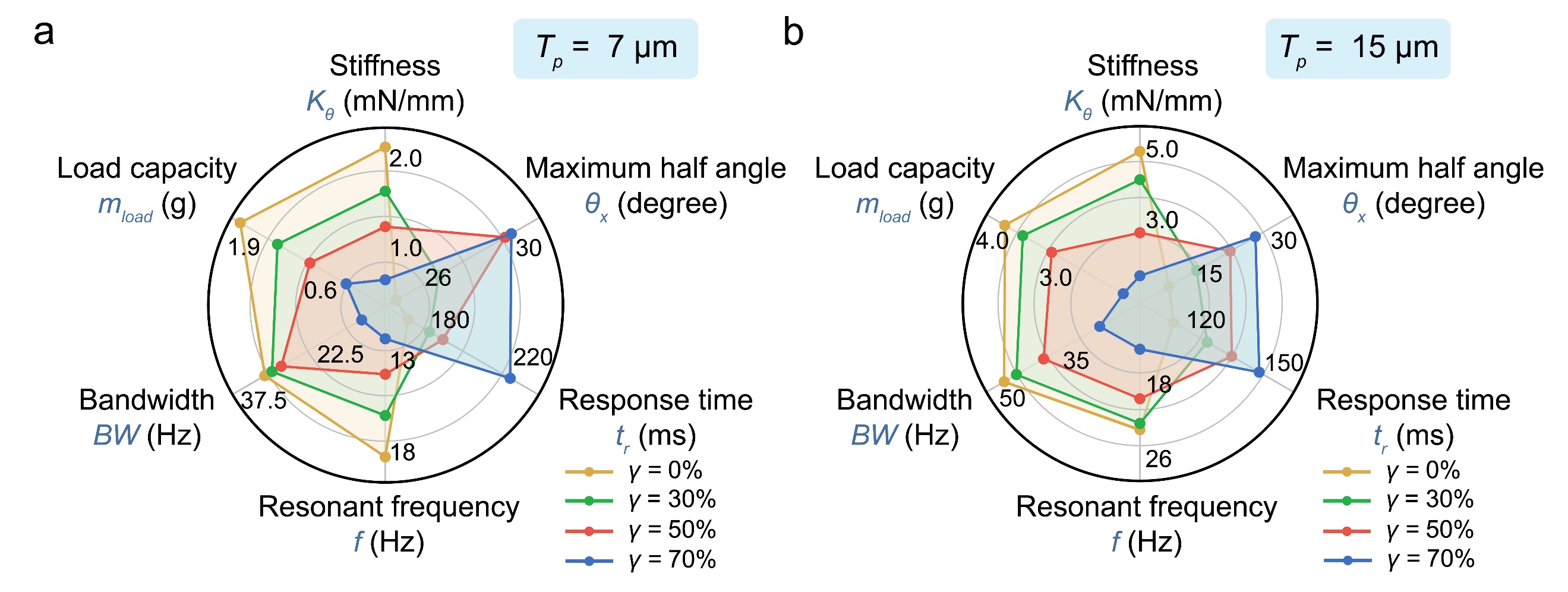


**Figure S4.** Multi-objective parametric optimization geometric of parameter *γ*, considering film thickness *t_p_*. **a,** *t_p_* =7 μm; **b,** *t_p_* =15 μm.

**Figure S4b** demonstrates that as *γ* progressively increases, the multidimensional mechanical performance trends for *t_p_* =15 μm align with those at *t_p_* =7 μm: rotational angle increases while load capacity and stiffness attenuate, and the rotational angle peaks at 28.2° when *γ*=70%. However, considering practical application priorities, where the FOV adjustment range supersedes other metrics, we selected the *t_p_* = 7μm with *γ*=50% parameter set to ensure angular displacements satisfy *θ_x_*, *θ_y_* beyond ±30°.

**Section SE:**

**Long-term mechanical and motion reliability evaluation of BES**

To evaluate the long-term mechanical and kinematic reliability of the BES, tests were conducted at two levels: the component level and the system level. First, a flexural deformation test was performed on the basic flexible unit of the MOM (rigid link–flexible hinge–rigid link), followed by a long-term assessment of the kinematic performance of the entire BES system.

**1.1 Long-term mechanical behavior testing of the basic flexible unit**

During system operation, the most common working condition faced by the flexible unit is deflection deformation under external force. To evaluate its long-term performance, we conducted a test in which one rigid link of the flexible unit was fixed, and an external force was applied to the other link while measuring the resulting angular deflection (**Figure S5a**). The test was performed under two conditions: one with the basic flexible unit without prior cyclic folding, and the other after the unit had undergone 36,000 reciprocal folding cycles within a 150° range (at 1 Hz for 5 hours).

As shown in **Figure S5b**, the angular deflection of the basic flexible unit increases nonlinearly with the applied force, primarily due to the reduction in effective moment arm length as the angle increases. Although slight differences are observed in the force-angle deflection curves before and after testing, these variations remain within an acceptable range. This relative stability in mechanical performance is a prerequisite for achieving high reliability in the BES system.


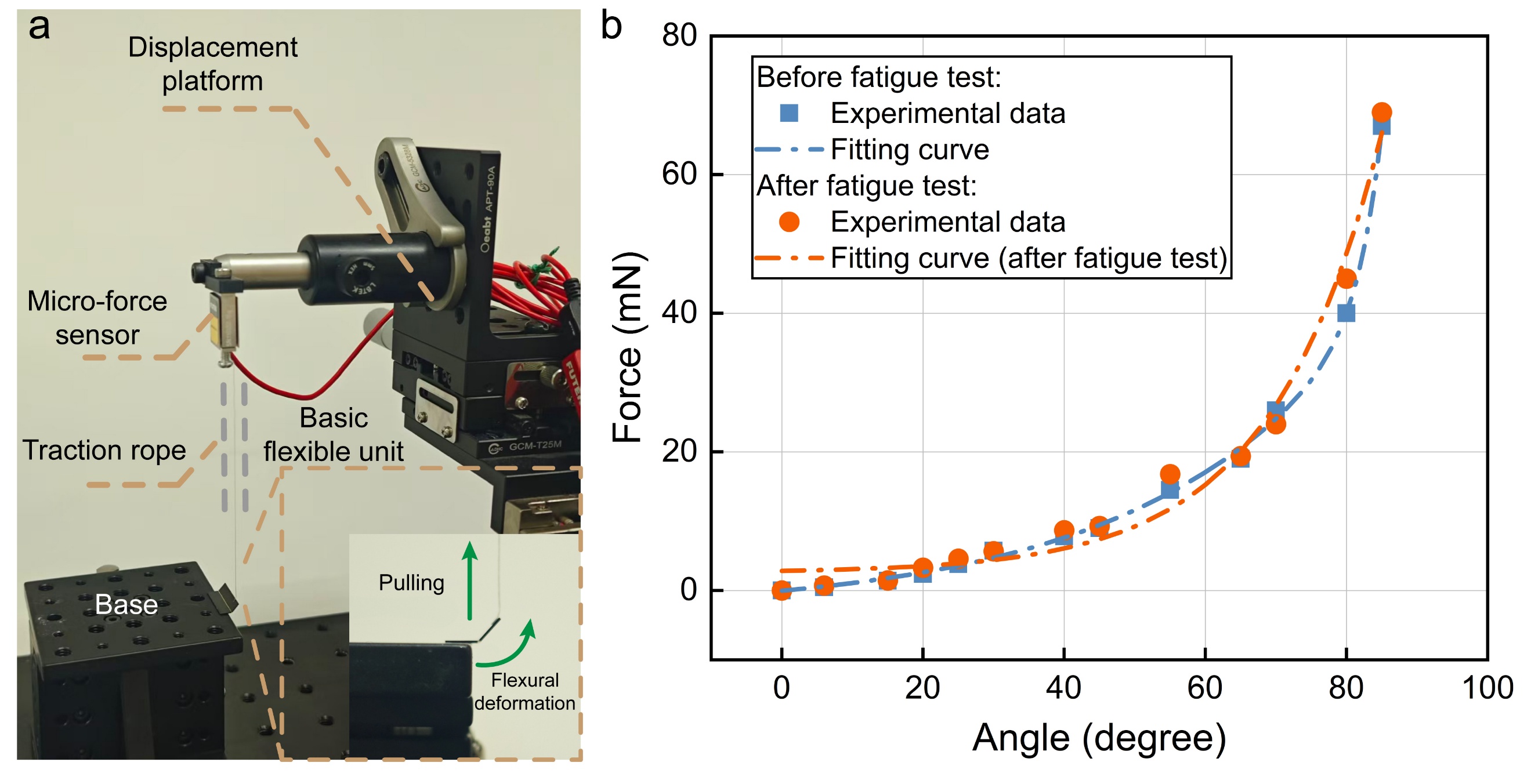


**Figure S5. Evaluation of the long-term mechanical behavior of the basic flexible unit. a,** Experimental setup of the flexural deformation test performed on the basic flexible unit using a micro-force sensor; **b,** Force–angular deflection curves obtained before and after the cyclic bending test.

**1.2 The motion reliability evaluation of BES**

To evaluate the kinematic reliability of the BES, a continuous operation test was conducted under Mode 2 with a duration of 3 hours and a frequency of 1 Hz. The results are shown in **Figure S6**. It can be observed that the output angle *θ_x_* exhibits certain fluctuations over time. However, a comparison of the output waveforms at the 3 s and 2.5 hours marks indicates that the amplitude of these fluctuations remains within an acceptable range and is consistent with the data measured in **Figure 3a** of the main text. This motion reliability stems primarily from two factors: the mechanical stability of the flexible hinge under long-term cyclic folding, as discussed in the previous section, and the output stability of the piezoelectric actuator due to its low current-induced thermal effects during operation.

**
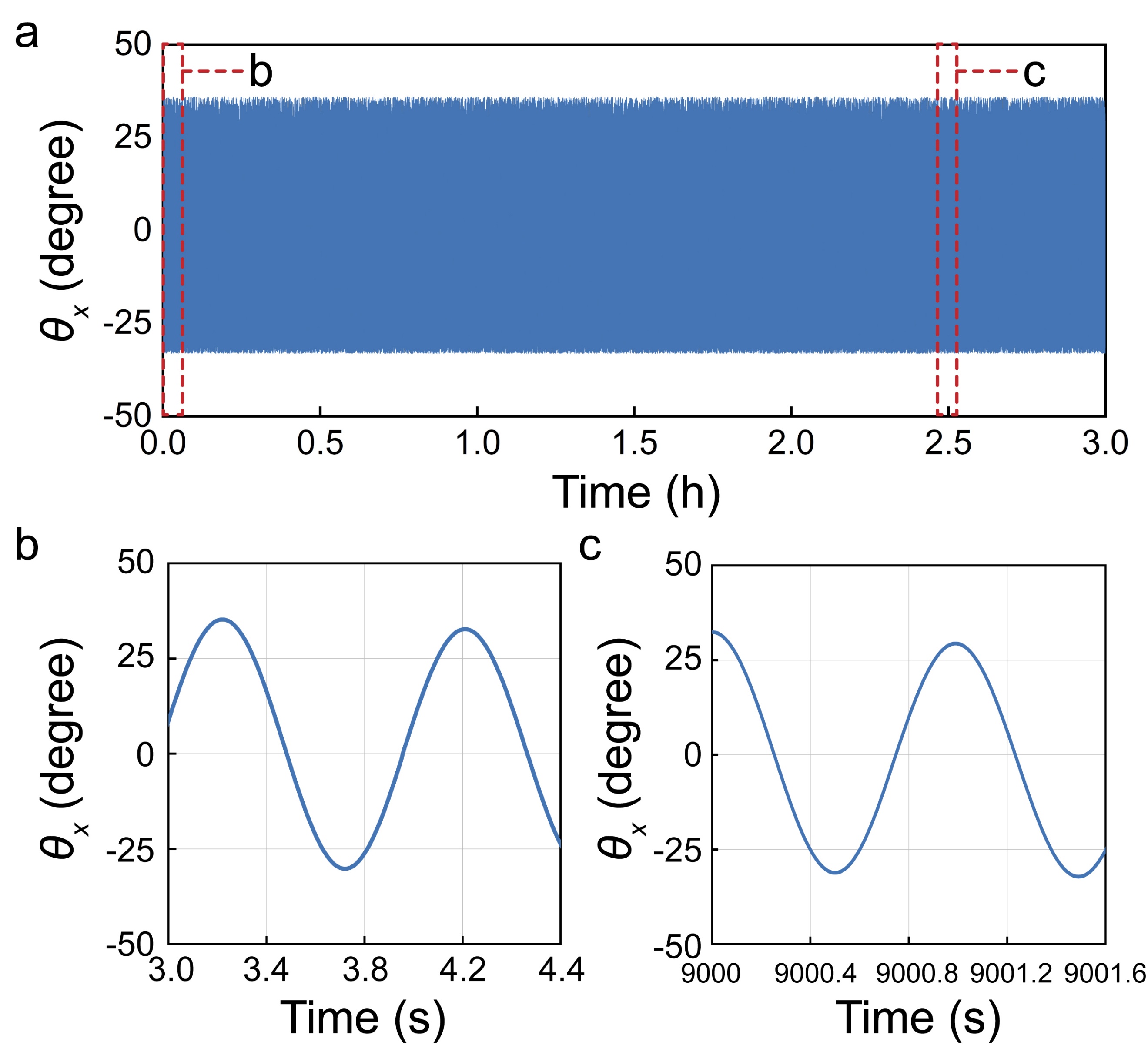
**

**Figure S6. Evaluation of the BES motion stability. a,** Angular displacement test of the BES under Mode 2, driven at 1 Hz for 3 hours; **b,** Angular displacement curve from 3 s to 4.4 s; **c,** Angular displacement curve from 9000 s (2.5 hours) to 9001.6 s.


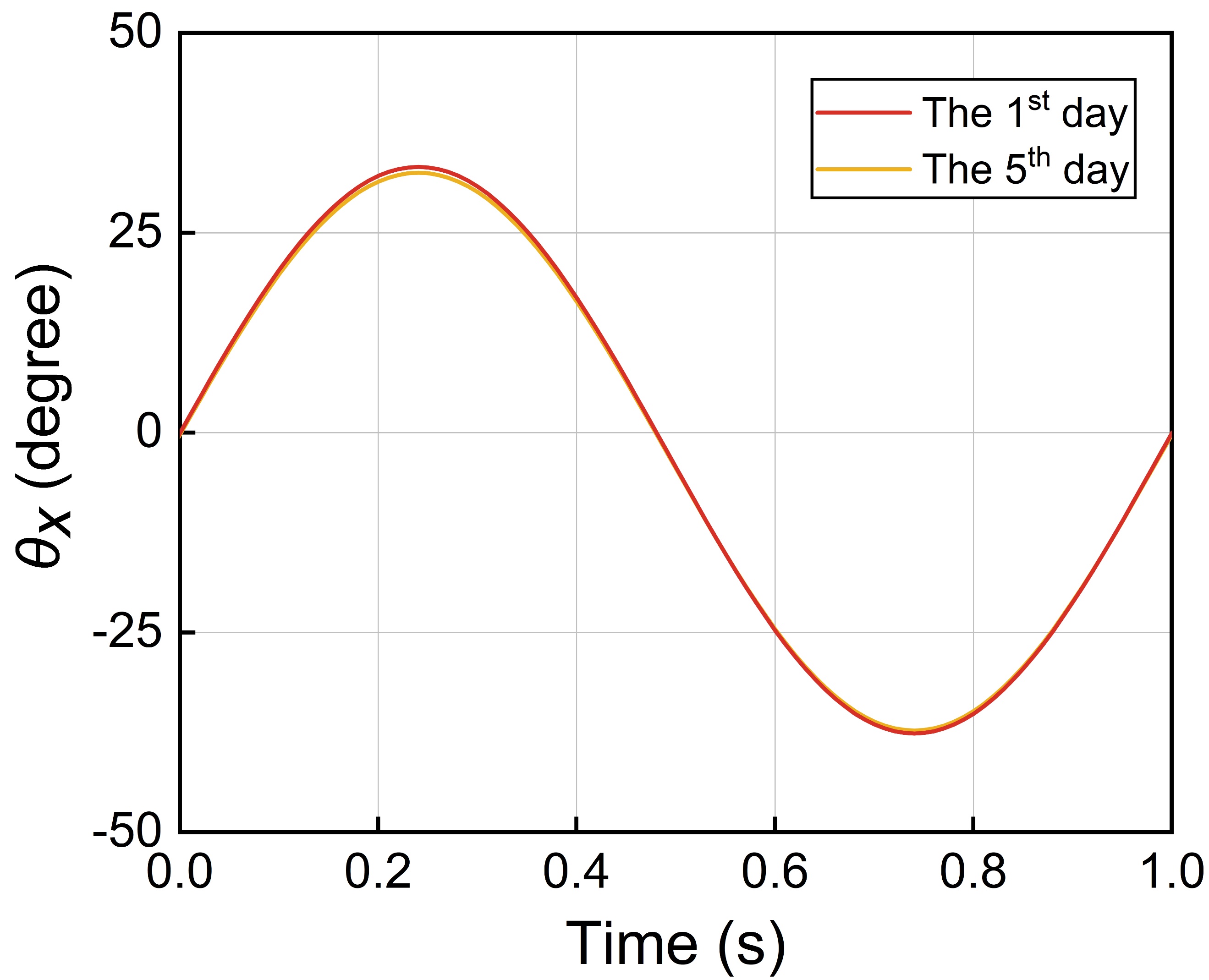


**Figure S7. Evaluation of the BES motion** **repeatability.**

Furthermore, to evaluate motion repeatability, two tests were conducted with a 5-day interval. The experimental results (**Figure S7**) demonstrate that the output of the BES remains highly consistent, further confirming the long-term stability of its performance and thereby enhancing the feasibility of this system for practical robotic applications.

**Section SF:**

**Impact and vibration resistance performance of BES**

Robust resistance to external vibration is critical for the BES to operate in robotic environments. To this end, this section presents a preliminary assessment of its impact and vibration resistance. As shown in **Figure S8**, the BES was mounted on a vibration platform that provided excitation along the vertical direction.


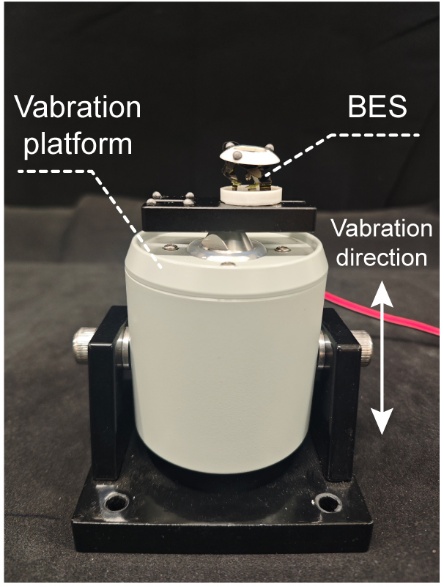


**Figure S8. Experimental setup of the impact and vibration response test.**

**1.1 Impact response test**

Firstly, impact resistance tests were conducted on the BES in its non-operational state. The shock input from the vibration platform was a square wave signal with a pulse width of 5 ms and a period of 5 s. As shown in **Figure S9**, the response signal amplitude of the BES rotating plane exceeded the vibration platform input by 20.8%.


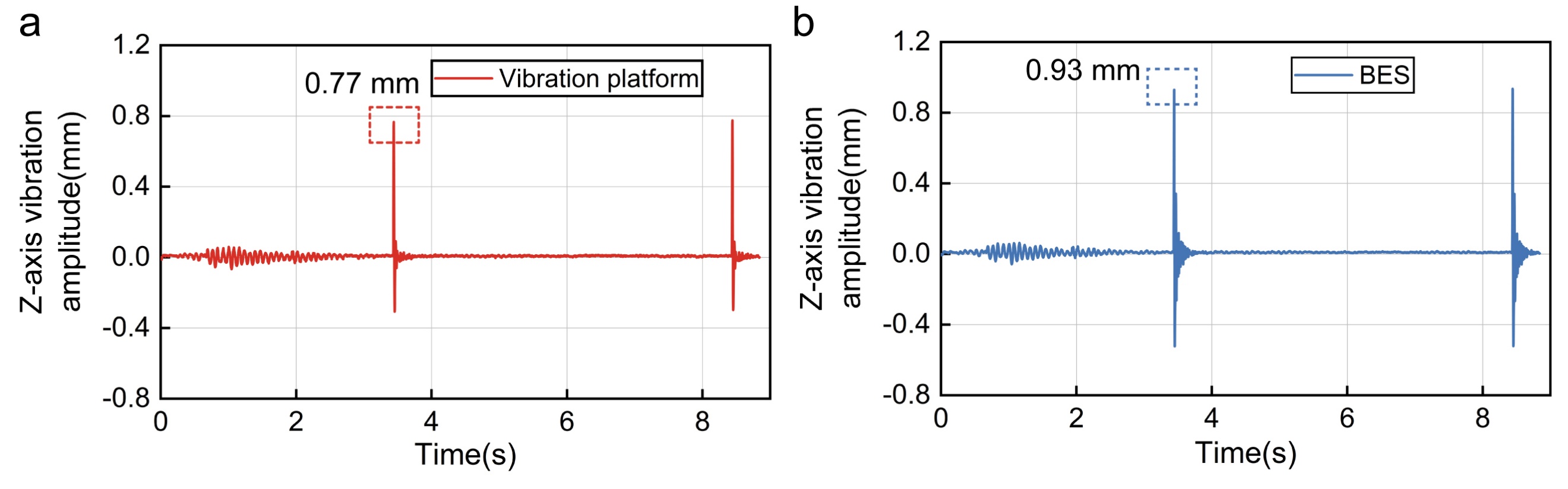


**Figure S9. Impact response test of the BES in its non-operational state.**

**1.2 Vibration test**

Then, the response of the BES under Mode 2 to continuous external vibration was tested, as shown in **Figure S10**. A sinusoidal driving signal at 1 Hz was applied to the BES (open-loop control), whereas the vibration platform was operated with square wave inputs at frequencies of 1 Hz, 5 Hz, and 10 Hz.

As the vibration amplitude increased from 0.5 mm to 1.5 mm, its disruptive effect on the single DOF rotation angle of the BES became more pronounced. Under the most severe condition (10 Hz, 1.5 mm), the rotational signal exhibited noticeable noise in the time domain. Despite this, the overall waveform remained clear, with a signal-to-noise ratio of 44.99 dB, indicating good signal integrity.

The anti-interference capability of the BES under low-frequency vibration can be enhanced through feedback control based on multi-sensor data fusion, along with improvements in passive damping and structural stiffness. These developments will support its evolution into an opto-mechatronic stabilization platform competitive with micro gimbals.


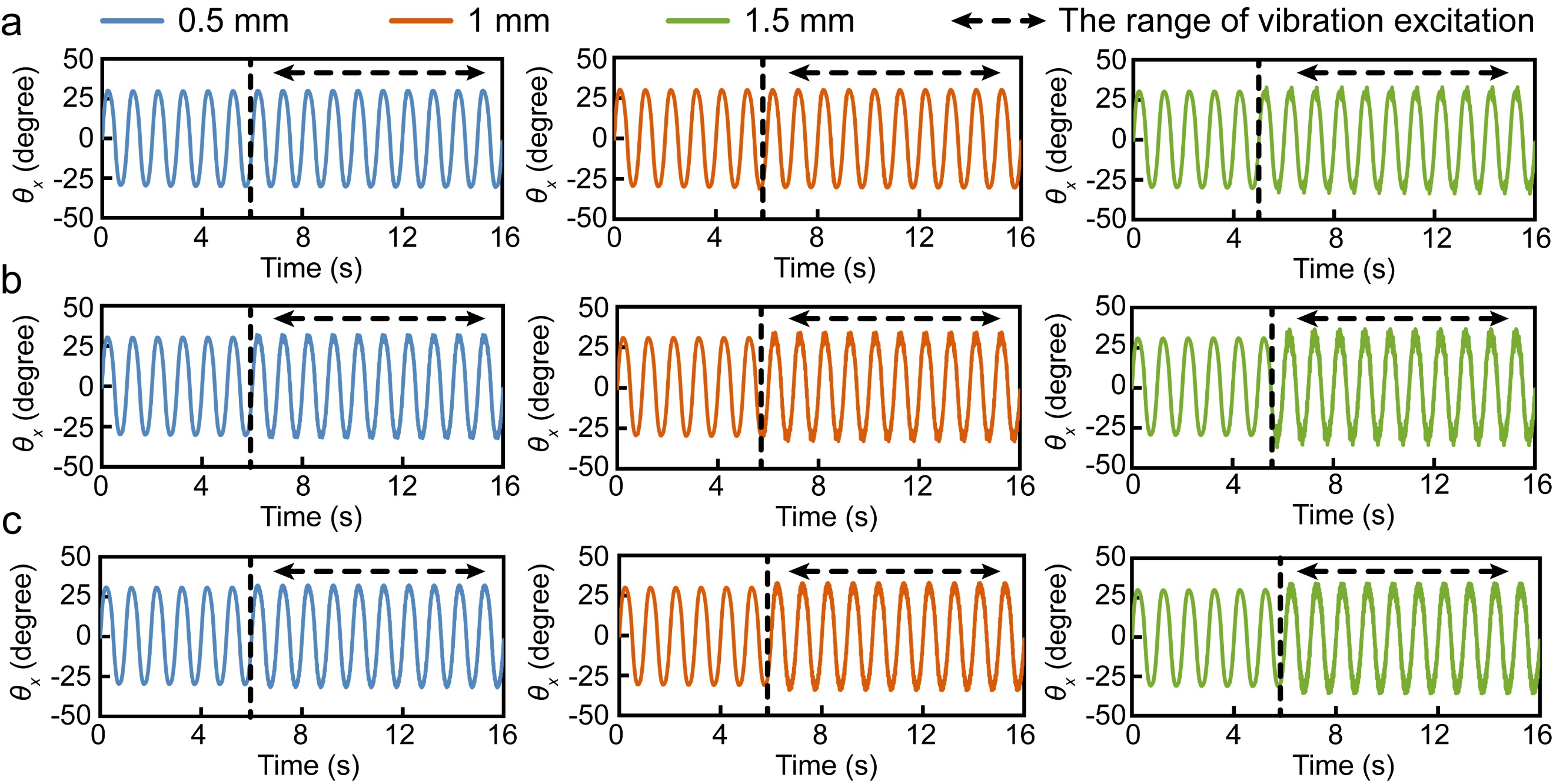


**Figure S10. Vibration test of the BES operating in Mode 2.** The Z-axis vibration frequencies of vibration plane are set as **a,** 1 Hz, **b,** 5 Hz, and **c,** 10 Hz.

**Table S1. SNR level during the vibration test.**

| Amplitude (mm)  Frequency (Hz) | 0.5 | 1 | 1.5 |
| --- | --- | --- | --- |
| 1 | 60.17 dB | 59.74 dB | 59.19 dB |
| 5 | 54.89 dB | 51.36 dB | 47.73 dB |
| 10 | 53.78 dB | 48.16 dB | 44.99 dB |

**Section G:**

**Kinematic and mechanical modeling of miniature origami mechanism (MOM)**

An in-depth analysis of the mechanical characteristics of the BES requires kinematic and mechanical models of the rigid-flexible composite micro-mechanism. To this end, this section aims to establish a set of modeling methodologies to guide the performance analysis and optimization design of the system. First, based on the geometric features of the MOM, an analytical framework for the passive rotation stage (PRS) is established, and the fully rigid kinematic model of the system is derived. Subsequently, a pseudo-rigid-body (PRB) model is introduced to construct simplified kinematic and static analytical models for the rigid-flexible composite structure. Furthermore, partial dynamic characteristics of the system are analyzed using finite element software.

**1.1** **Geometric analysis of PRS**

The overall composition of the MOM includes the active driving platforms (ARSs) for rotational displacement input and PRS for achieving 3 DOFs decoupled motion. The structural design of the PRS is key to enabling large-range rotational and translational motions. Therefore, this section begins with a detailed analysis of the geometric design of the PRS, followed by the development of its theoretical kinematic model.

As shown in **Figure S11a**, the two-dimensional planar layout of the PRS mainly consists of a triangular rotation plane for angular displacement output and three motion branches responsible for driving displacement transmission, amplification, and decoupling. To maximize spatial efficiency, the three motion branch chains are externally connected to the three sides of the triangular plane with a 120° angular spacing.


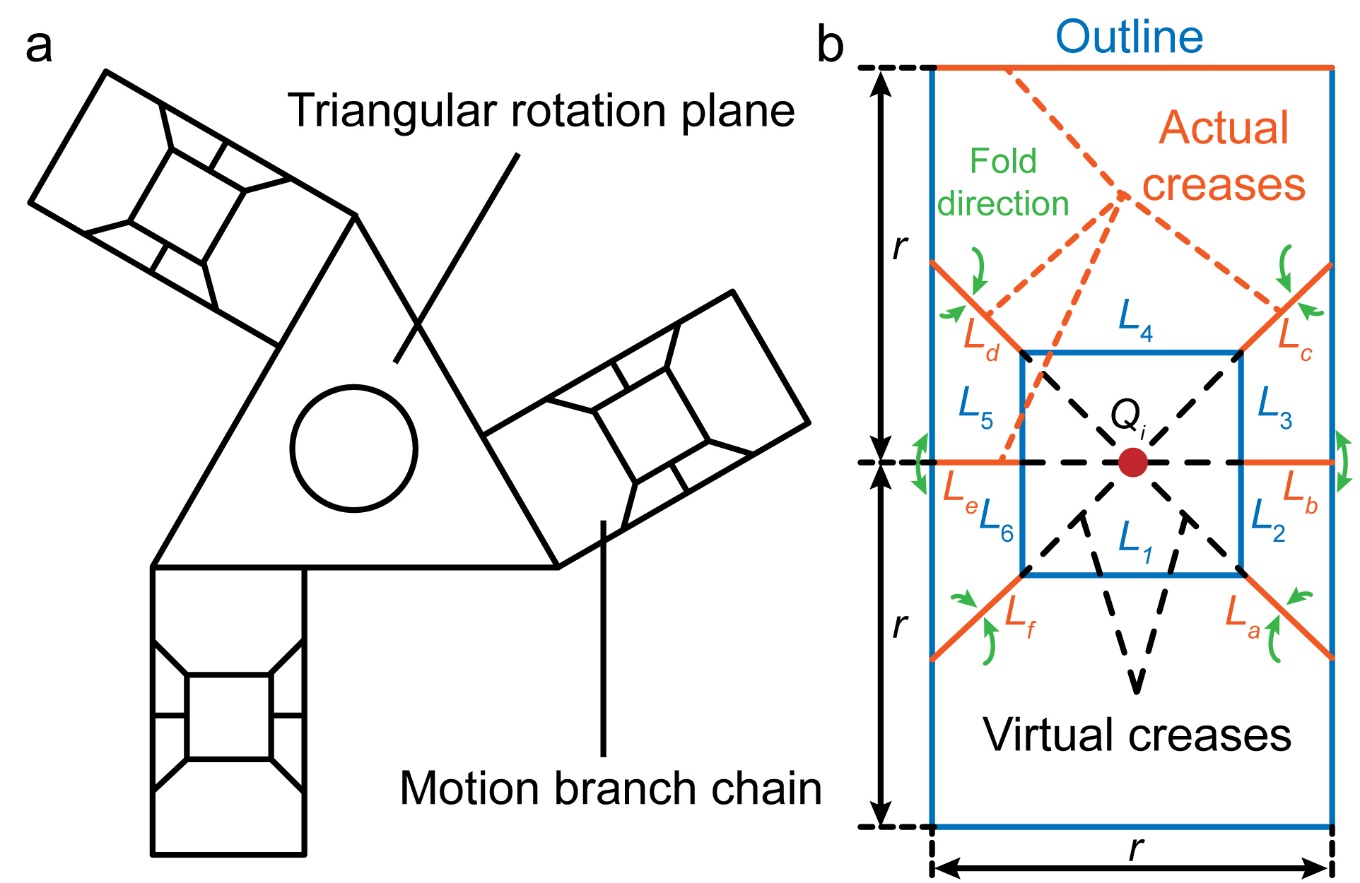


**Figure S11. 2D planar unfolding structure of PRS. a,** Overall unfolding structure; **b,** Unfolding structure of single motion branch chain.

The most critical structural components of the PRS are its three motion branches. The planar state of a single branch is illustrated in **Figure S11b**. It consists of seven actual creases, along with six virtual creases formed by extending the physical creases into the central hollow region. These virtual creases converge at a common point, *Q_i_*. Since PRS is rigidly integrated with ARSs via the three branches, and the output of the active platform is a single DOF angular displacement about the rotation axis, the lower contour of each branch can be equivalently regarded as an actual crease. As a result, the entire structure exhibits a total of 14 creases, which are centrally symmetric about the common point.

Folding the two-dimensional planar structure shown in **Figure S11b** along the creases into three-dimensional space yields the spherical 6R mechanism illustrated in **Figure S12**. The spatial configuration of this mechanism is primarily determined by the angles *α_i,j_* (*i*=1, 2, 3；*j*=1, 2, … , 6) between the six virtual creases. Since the structure in **Figure S12b** is a mapping of **Figure S11b** from 2D to 3D space, the angle values are set as *α_i_*_,1_ = *α_i_*_,4_ = 90° and *α_i_*_,2_ = *α_i_*_,3_ = *α_i_*_,5_ = *α_i_*_,6_ = 45°. This geometric relationship forms the dimensional basis for achieving central and geometric symmetry in each branch.

In addition to the virtual crease angles, the rotational angles *θ_i,j_* (i=1, 2, 3；j=1, 2, … , 6) of the equivalent links along the creases also collectively determine the spatial posture of the spherical 6R mechanism and the motion posture of the MOM under the coordinated movement of the three branches. As shown in **Figure S12c** and **Figure S12d**, when an external load causes angular displacement deflection in the branch along the indicated direction, the rotational angles *θ_i,j_* change significantly compared to those in **Figure S12b**, while the angles *α_i,j_* remain unchanged. The specific method for solving *θ_i,j_* will be detailed in the subsequent kinematic analysis.


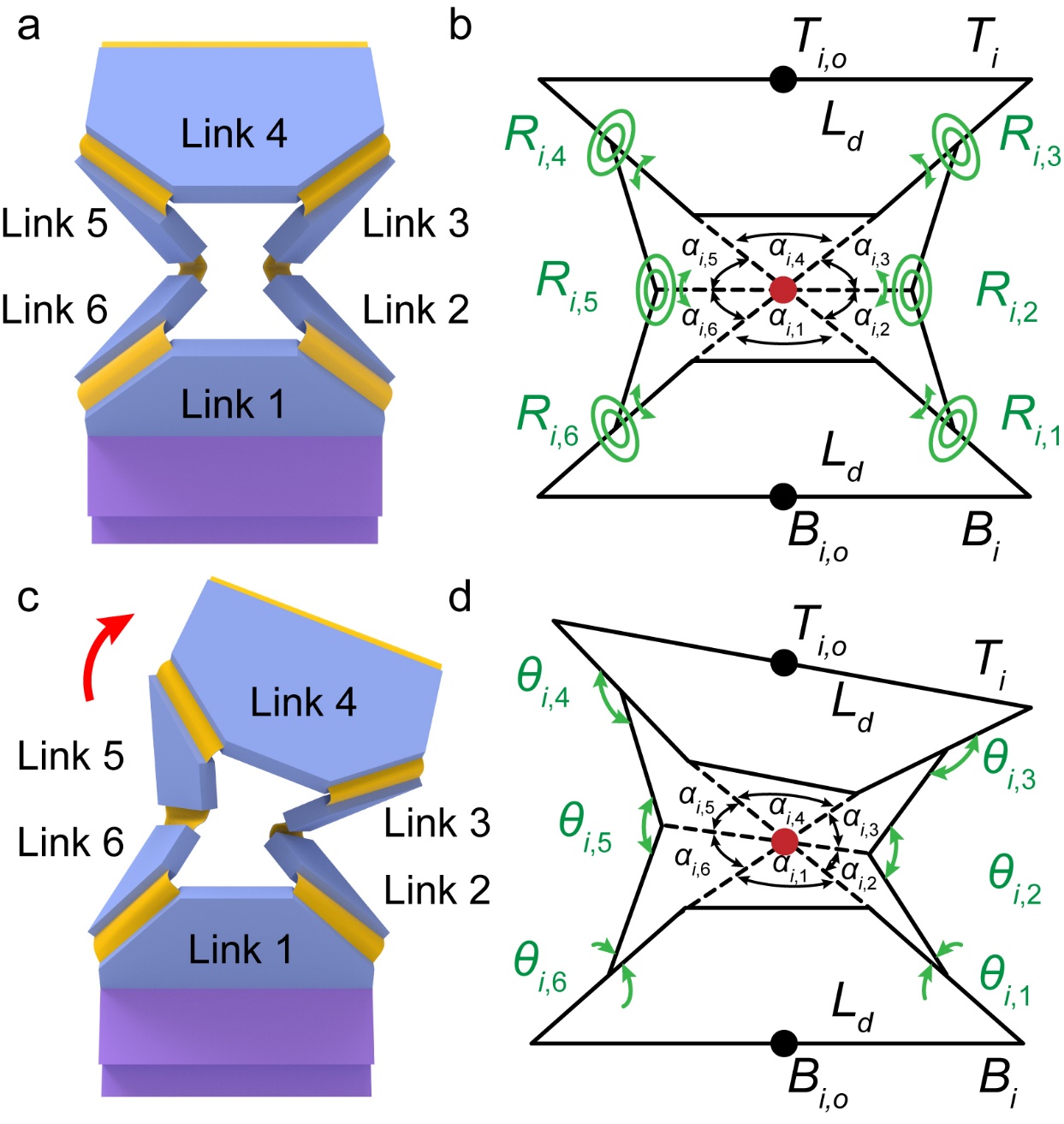


**Figure S12. 3D model and kinematic diagram of a single branch. a, b,** Initial state; **c, d,** State under external load-induced deflection (arrow direction).

As shown in **Figure S12**, Link 1 and Link 4 are connected to the angular displacement output end B_i_ (fixed to the ARS) and the outer edge *T_i_* of the triangular rotation plane. Thus, the common intersection point *Q_i_* can be regarded as performing arc motions around *B_i_*_,o_ (the midpoint of *B_i_*) and *T_i_*_,o_ (the midpoint of *T_i_*), respectively, with the same radius *r*, i.e., *Q_i_B_i,o_* = *Q_i_T_i,o_* = *r*. Based on this geometric relationship, the spatial pose of the rotation plane can be uniquely determined by *B_i,o_*, *Q_i_*, and *T_i,o_* at any given spatial position, which corresponds to the forward kinematics process. Conversely, the angular displacement output of the ARSs can be inversely derived from the three points *T_i,o_*, *Q_i_*, and *B_i,o_*, corresponding to the inverse kinematics process.

After clarifying the motion trajectory of the common intersection point *Q_i_* of a single branch relative to *B_i_* and *T_i_*, the geometric relationships during the motion of the PRS can be established, as shown in **Figure S13**. This figure illustrates the range of motion of the common intersection points *Q_i_* in the three branches when the system undergoes single DOF translational motion (where the fixed and moving platforms remain parallel and horizontal), represented by the colored dash-dotted lines.

Taking the branch *B*_1_,*_o_*–*Q*_1_,*_o_*–*T*_1_,*_o_* as an example, connect *B*_1_,*_o_T*_1_,*_o_*, and from the intersection point *Q*_1_ of the two arc trajectories, draw a perpendicular segment *Q*_1_*P*_1_ to *B*_1_,*_o_T*_1_,*_o_*, intersecting at *P*_1_. According to the congruent criterion of right angled triangles, Δ*Q*_1_*P*_1_*T*_1_,*_o_* ≌ Δ*Q*_1_*P*_1_*B*_1_,*_o_*, thus *P*_1_*T*_1_,*_o_* = *P*_1_*B*_1_,*_o_*.

Furthermore, based on the system's kinematic characteristics of having only two rotational DOFs and one translational degree of freedom, when the fixed and moving platforms are parallel, the moving platform must be directly above the fixed platform, meaning *B*_1_,*_o_T*_1_,*_o_* is perpendicular to the planes of both platforms. Therefore, the plane defined by Δ*Q*_1_*T*_1_,*_o_B*_1_,*_o_* is also perpendicular to the platform planes. Under this condition, the extension of *Q*_1_*P*_1_ must intersect the other arc intersection point *Q*_1_′, and the same applies to the remaining branches. Given that *P*_1_*T*_1_,*_o_* = *P*_1_*B*_1_,*_o_*, it can be deduced that the triangle formed by the three perpendicular segments *Q*_1_*Q*_1_′, *Q*_2_*Q*_2_′, and *Q*_3_*Q*_3_′ is parallel to the fixed and moving platform planes, and its plane α is the symmetry plane of the two platform planes.


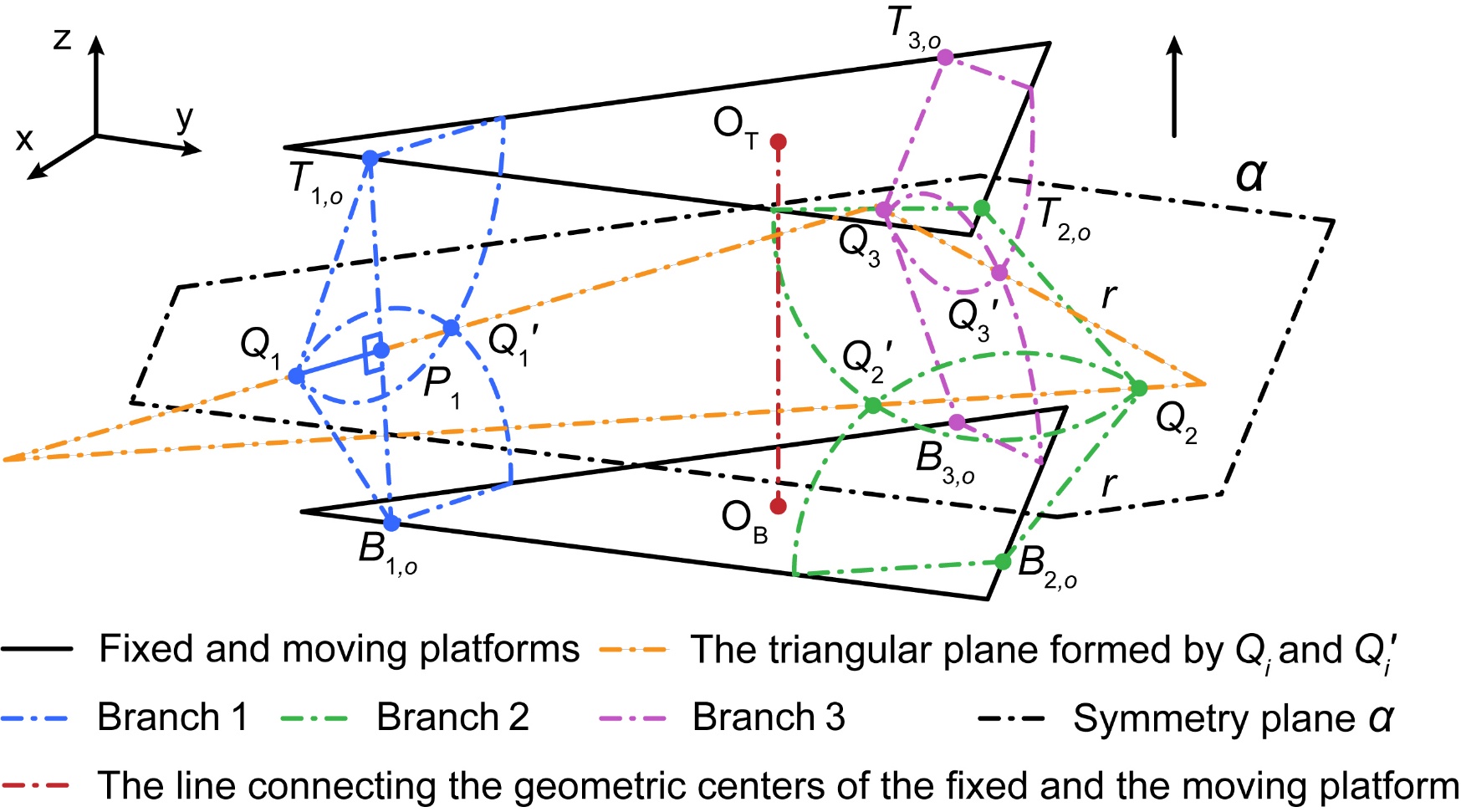


**Figure S13.** **Geometric relationships during the motion of the PRS under a single DOF translational motion (with the moving and fixed platforms remaining parallel).**

When the moving platform undergoes deflection, the posture changes of the three branches are illustrated in **Figure S14**. At this point, *B_i,o_T_i,o_* is angularly offset relative to the fixed platform, so Δ*Q_i_T_i,o_Q_i_*′ and Δ*Q_i_B_i,o_Q_i_*′ no longer lie in the same plane.

Taking the branch *B*_1_,*_o_*–*Q*_1,o_–*T*_1,o_ as an example, connect *B*_1,o_*T*_1,o_, *T*_1,o_*Q*_1_′, and *B*_1,o_*Q*_1_′. From point *Q*_1_, draw a perpendicular segment *Q*_1_*P*_1_ to *B*_1_,*_o_T*_1_,*_o_*, intersecting at *P*_1_, and then connect *P*_1_*Q*_1_′. Based on the previous analysis, Δ*Q*_1_*P*_1_*T*_1_,*_o_* ≌ Δ*Q*_1_*P*_1_*B*_1_,*_o_*, hence *P*_1_*T*_1_,*_o_* = *P*_1_*B*_1_,*_o_*. It can be further proven that Δ*Q*_1_′*P*_1_*T*_1_,*_o_* ≌ Δ*Q*_1_′*P*_1_*B*_1_,*_o_* (corresponding sides are equal), and thus *Q*_1_′*P*_1_ is also perpendicular to *B*_1_,*_o_T*_1_,*_o_*.

Since *B*_1_,*_o_T*_1_,*_o_* is perpendicular to both *Q*_1_*P*_1_ and *Q*_1_′*P*_1_, it is perpendicular to any line within the plane formed by them, meaning *B*_1_,*_o_T*_1_,*_o_* ⟂ *Q*_1_*Q*_1_′. The same reasoning applies to the other two branches: *B*_2_,*_o_T*_2_,*_o_* ⟂ *Q*_2_*Q*_2_′ and *B*_3_,*_o_T*_3_,*_o_* ⟂ *Q*_3_*Q*_3_′.

By connecting *T*_1_,*_o_T*_2_,*_o_*, *B*_1_,*_o_B*_2_,*_o_*, and *P*_1_*P*_2_, the geometric relationships indicate that *P*_1_*P*_2_ is a common perpendicular segment to the top and bottom bases of the isosceles trapezoid *T*_1_,*_o_T*_2_,*_o_B*_2_,*_o_B*_1_,*_o_*. The same applies to branch 3 (not shown in **Figure S14**). Therefore, *B_i,o_T_i,o_* is perpendicular to both *P_i_P_j_* (*j*≠*i*) and *P_i_P_k_* (*k*≠*i*，*k*≠*j*), and is bisected by *P_i_P_j_* and *P_i_P_k_*.

In summary, the plane defined by Δ*P*_1_*P*_2_*P*_3_ perpendicularly bisects each *B_i,o_T_i,o_*, meaning it is the symmetry plane of the fixed and moving platforms.

Based on the fact that *B_i,o_T_i,o_* is perpendicular to both the plane *Q*_i_*Q*_i_′*P_i_* and the plane *P*_1_*P*_2_*P*_3_, it can be deduced that these two planes are either parallel or coplanar. Since they share a common point P_i_, they must be coplanar, meaning the line segment *Q_i_Q_i_*′ lies within the plane *P*_1_*P*_2_*P*_3_. Therefore, the symmetry plane formed by Δ*P*_1_*P*_2_*P*_3_ can be extended to the plane α defined by the three line segments *Q*_1_*Q*_1_′, *Q*_2_*Q*_2_′, and *Q*_3_*Q*_3_′.


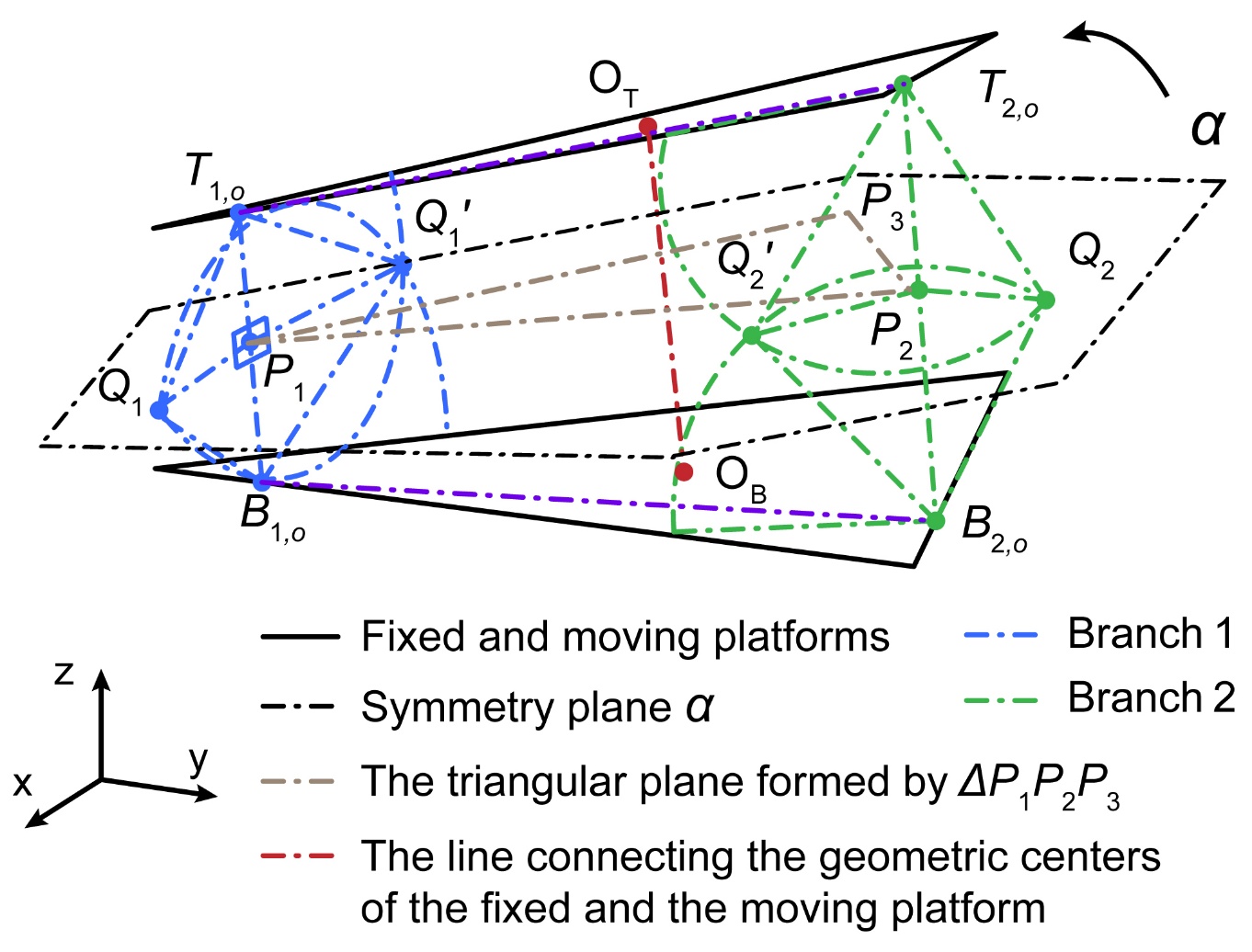


**Figure S14.** **Geometric relationships during the motion of the PRS under the rotational motion.**

In summary, regardless of whether the system is in a translational or rotational motion state, there invariably exists a symmetry plane α about which the moving platform and the fixed platform exhibit mirror symmetry. This plane α is uniquely determined by the spatial positions of the common intersection points *Q_i_* of the three motion branches.

**1.2 Kinematic analysis based on multi-rigid body models of MOM**

**1.2.1 Kinematic analysis of PRS**

**1.2.1.1 Forward kinematics**

The forward kinematic solution strategy for the PRS can be formulated as follows: given the angular displacement inputs of the three motion branches driven by the ARSs, determine the posture of the moving platform or the position of any point on it.

After clarifying the geometric relationships during the motion of the PRS, the kinematic model shown in **Figure S15** can be established. A global coordinate system O_B_-XYZ is defined at the geometric center O_B_ of the fixed platform, where the X-axis is perpendicular to segment AB, the Z-axis is vertical upward, and the Y-axis direction is determined by the right-hand rule. Additionally, a local coordinate system *B_i,o_-x_i_y_i_z_i_* is established at *B_i,o_* of each branch, where the *x_i_*-axis is perpendicular to *B_i_*, the *z_i_*-axis is vertical upward, and the *y_i_*-axis direction is determined by the right-hand rule.

For the common intersection point *Q_i_* in the branch *B_i,o_–Q_i,o_–T_i,o_*, let the angle between the line *B_i,o_Q_i_* and the *x_i_*-axis be *θ_i,B_*. Then, the coordinates of *Q_i_* in the local coordinate system *B_i,o_-x_i_y_i_z_i_* can be expressed as:

|  | $Q_{i}^{'}=\left[\begin{matrix} r\cos\theta_{i,B} & 0 & r\sin\theta_{i,B} & 1 \end{matrix}\right]^{\text{T}}$ | (1) |
| --- | --- | --- |

The position of *Q_i_* in the global coordinate system can be represented by a coordinate transformation matrix:

|  | $Q_{i}=\mathbf{R}_{Z,i}^{-1}\mathbf{T}_{XY,i}^{-1}Q_{i}^{'}$ | (2) |
| --- | --- | --- |


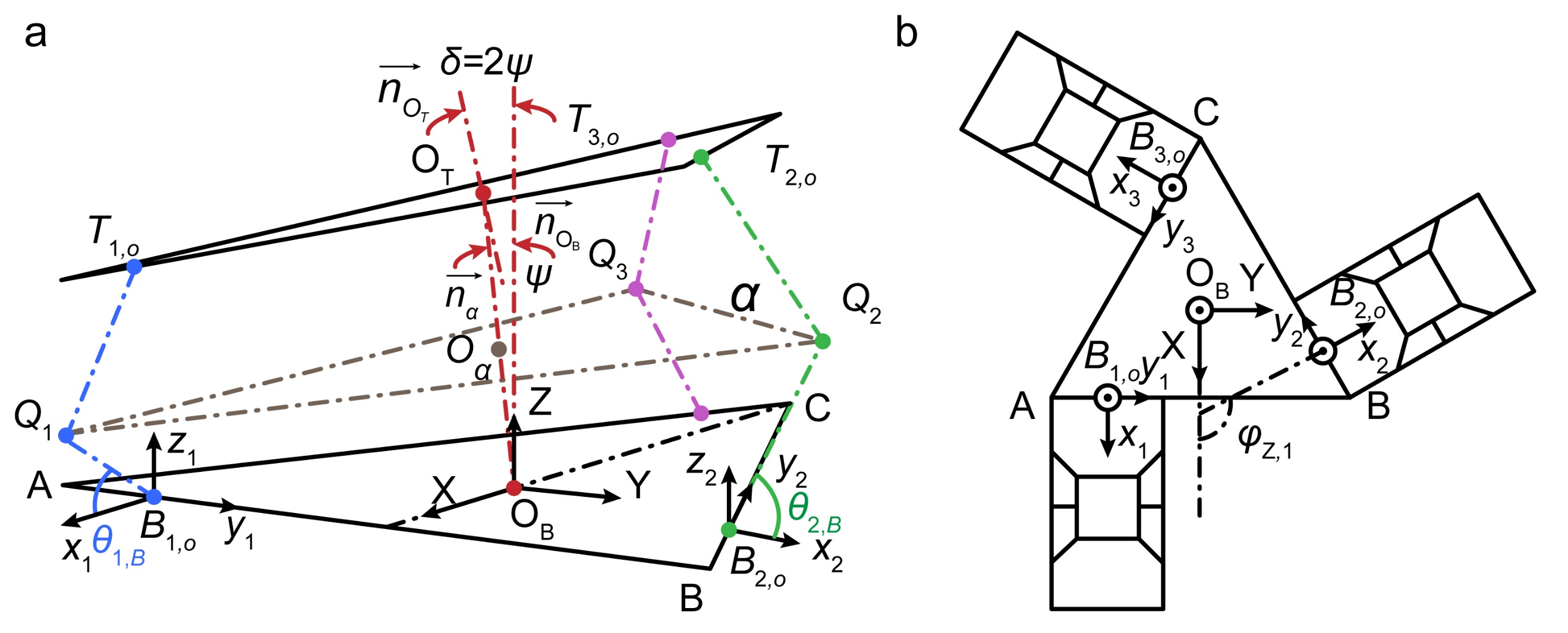


**Figure S15. Kinematic model of the PRS. a,** 3D spatial view; **b,** 2D planar view (from the fixed platform).

where $\mathbf{T}_{XY,i}^{-1}$ and $\mathbf{R}_{Z,i}^{-1}$ are the inverse matrices of the translation transformation matrix and rotation transformation matrix from the global coordinate system *O_B_-XYZ* to the local coordinate system *B_i,o_-x_i_y_i_z_i_* respectively:

|  | $\mathbf{T}_{XY,i}=\left[\begin{matrix} 1 & 0 & 0 & T_{X,i} \\ 0 & 1 & 0 & T_{Y,i} \\ 0 & 0 & 1 & T_{Z,i} \\ 0 & 0 & 1 & 1 \end{matrix}\right]$ | (3) |
| --- | --- | --- |
|  |  | (4) |

The specific parameters in the equation are listed in **Table S2**, where *l_a_*​ denotes the side length of the triangular plane. By combining equations (2) to (4), the coordinates of *Q_i_* in the global coordinate system *O_B_-XYZ* can be expressed as:

|  |  | (5) |
| --- | --- | --- |

**Table S2. Parameters in the coordinate rotation transformation matrix.**

| Transformation matrixes | Parameters | Values |
| --- | --- | --- |
| ***T****_XY,i_* | *T_X,i_* | -$\frac{\sqrt{3}}{6}l_{a}$ |
|  | *T_Y,i_* | $\frac{1}{2}\left(l_{a}-r\right)$ |
|  | *T_Z,i_* | 0 |
| ***R****_Z,i_* | *φ_Z_,*_1_ | 0 |
|  | *φ_Z_,*_2_ | -$\frac{2\text{π}}{3}$ |
|  | *φ_Z_,*_3_ | -$\frac{4\text{π}}{3}$ |

According to the principle of planar mirror symmetry, the line (O_B_*O_T_*) connecting the geometric centers of the two mirror-symmetric planes must be perpendicular to the symmetry plane α. Therefore, the nonhomogeneous normal vector ($\overrightarrow{n_{\alpha}}$) of plane α can be expressed as:

|  | $\overrightarrow{n_{\alpha}}=\left(a,b,c\right)=\overrightarrow{\text{O}_{\text{B}}O_{T}}$ | (6) |
| --- | --- | --- |

Construct two vectors within the plane α:

$\overrightarrow{Q_{1}Q_{2}}=\left(x_{2}-x_{1},y_{2}-y_{1},z_{2}-z_{1}\right)$ *and $\overrightarrow{Q_{1}Q_{3}}=\left(x_{3}-x_{1},y_{3}-y_{1},z_{3}-z_{1}\right)$, and then,*

|  | $\left\{\begin{matrix} a=\left(y_{2}-y_{1}\right)\left(z_{3}-z_{1}\right)-\left(z_{2}-z_{1}\right)\left(y_{3}-y_{1}\right) \\ b=\left(z_{2}-z_{1}\right)\left(x_{3}-x_{1}\right)-\left(x_{2}-x_{1}\right)\left(z_{3}-z_{1}\right) \\ c=\left(x_{2}-x_{1}\right)\left(y_{3}-y_{1}\right)-\left(y_{2}-y_{1}\right)\left(x_{3}-x_{1}\right) \end{matrix}\right.$ | (7) |
| --- | --- | --- |

In the equation, the position coordinates of *Q*_1_ (*x*_1_, *y*_1_, *z*_1_) and *Q*_2_ (*x*_2_, *y*_2_, *z*_2_) can be calculated according to equation (5). Once this is determined, an equation can be formulated using the property that the inner product of any line within the plane and the normal vector of the plane is zero:

|  | $\overrightarrow{n_{\alpha}}\cdot\overrightarrow{O_{\alpha}Q_{1}}=\overrightarrow{n_{\alpha}}\cdot\left(\overrightarrow{\text{O}_{\text{B}}Q_{1}}-\overrightarrow{\text{O}_{\text{B}}O_{\alpha}}\right)=\mathbf{0}$ | (8) |
| --- | --- | --- |

namely,

|  | $\left[\begin{matrix} a & b & c \end{matrix}\right]\cdot\left[\begin{matrix} x_{1} \\ y_{1} \\ z_{1} \end{matrix}\right]-\left\vert\overrightarrow{\text{O}_{\text{B}}O_{\alpha}}\right\vert=0$ | (9) |
| --- | --- | --- |
|  | $\left\vert\overrightarrow{O_{\alpha}\text{O}_{\text{B}}}\right\vert=ax_{1}+by_{1}+cz_{1}$ | (10) |

where *O_α_* is the intersection point of the normal vector $\overrightarrow{n_{\alpha}}$ passing through O_B_ and the plane α. Thus,

|  | $\left\vert\overrightarrow{\text{O}_{\text{B}}O_{T}}\right\vert=2\times\left\vert\overrightarrow{O_{\alpha}\text{O}_{\text{B}}}\right\vert=2\left(ax_{1}+by_{1}+cz_{1}\right)$ | (11) |
| --- | --- | --- |

Let the global coordinates of point *O_T_* be *O_T_* (*x_T_*, *y_T_*, *z_T_*),

|  | $\overrightarrow{\text{O}_{\text{B}}O_{T}}=\lambda\overrightarrow{n_{\alpha}}=\lambda\left(a,b,c\right)$ | (12) |
| --- | --- | --- |
|  | $\left\vert\overrightarrow{\text{O}_{\text{B}}O_{T}}\right\vert=\lambda\sqrt{a^{2}+b^{2}+c^{2}}=2\left(ax_{1}+by_{1}+cz_{1}\right)$ | (13) |
|  | $\lambda=\frac{2\left(ax_{1}+by_{1}+cz_{1}\right)}{\sqrt{a^{2}+b^{2}+c^{2}}}$ | (14) |

Thus,

|  | $\overrightarrow{\text{O}_{\text{B}}O_{T}}=\frac{2\left(ax_{1}+by_{1}+cz_{1}\right)}{\sqrt{a^{2}+b^{2}+c^{2}}}\left(a,b,c\right)$ | (15) |
| --- | --- | --- |

The global coordinates of point *O_T_* have been determined. To solve for the plane equation of the moving platform, it is necessary to define the normal vector of this plane. Let *ψ* be the angle between the normal vector $\overrightarrow{n_{\text{O}_{\text{B}}}}$ of the fixed platform (i.e., the Z-axis direction of the global coordinate system) and the normal vector $\overrightarrow{n_{\alpha}}$of the symmetry plane α. Then:

|  | $\psi=\arccos\left(\frac{c}{\sqrt{a^{2}+b^{2}+c^{2}}}\right)$ | (16) |
| --- | --- | --- |

It also follows from the principle of planar mirror symmetry that the angle between the normal vector of moving platform $\overrightarrow{n_{\text{O}_{T}}}$ and the global Z-axis is 2*ψ*. Thus, the vector is determined as $\overrightarrow{n_{\text{O}_{T}}}=\left(j,k,l\right)$:

|  | $\left\{\begin{matrix} j=a \\ k=b \\ l=\frac{\sqrt{j^{2}+k^{2}}}{\tan2\psi} \end{matrix}\right.$ | (17) |
| --- | --- | --- |

With the global coordinates of the geometric center of the moving platform and its normal vector at that point now determined, the plane equation of the moving platform can be expressed as:

|  | $j\left(x-\lambda a\right)+k\left(y-\lambda b\right)+l\left(z-\lambda c\right)=0$ | (18) |
| --- | --- | --- |
|  | $jx+ky+lz-\lambda\left(ja+kb+lc\right)=0$ | (19) |

Thus, the forward kinematic solution for PRS has been established. Using the mathematical model described above, the coordinates of any point on the moving platform during its motion can be solved. For example, to determine the coordinate change from point *N* (*x_N_*, *y_N_*, *z_N_*) to point *N*′ (*x_N_*′, *y_N_*′, *z_N_*′) when the platform undergoes rotational motion from a horizontal position, the following system of equations can be formulated:

|  | $\left\{\begin{matrix} jx_{N}^{'}+ky_{N}^{'}+lz_{N}^{'}-\lambda\left(ja+kb+lc\right)=0 \\ \left(x_{N}^{'}-\lambda a\right)^{2}+\left(y_{N}^{'}-\lambda b\right)^{2}+\left(z_{N}^{'}-\lambda c\right)^{2}=x_{N}^{2}+y_{N}^{2} \\ \frac{y_{N}}{x_{N}}=\frac{y_{N}^{'}-\lambda b}{x_{N}^{'}-\lambda a} \end{matrix}\right.$ | (20) |
| --- | --- | --- |

The physical meaning of the equation system (20) is as follows:

1) The first equation defines the plane equation of the moving platform on which point *N*′ lies.

2) The second equation specifies that the distance between *N*′ and *O_T_* equals the distance between *N* and *O_B_*.

3) The third equation ensures that the azimuth angle of *N*′ on the moving plane equals the azimuth angle of *N* on the fixed plane.

Solving the equation system (20) yields two solutions. One of them is an extraneous solution corresponding to a symmetrically opposite point *N*′′ (*x_N_*′′，*y_N_*′′，*z_N_*′′) with respect to *O_T_*. The valid solution can be identified by requiring that the sign of *x_N_*′ matches the sign of *x_N_*.

**1.2.1.2 Inverse kinematics**

For the inverse kinematics of the system, the solution strategy is to determine the active rotation angles *θ_i,B_* of the three branches based on the known position of the geometric center *O_T_ (x_T_, y_T_, z_T_)* of the moving platform.

Since *O_α_* is the midpoint of *O_B_O_T_*, its global coordinates is *O_α_* (0.5*x_T_*, 0.5*y_T_*, 0.5*z_T_*). Given that $\overrightarrow{\text{O}_{\text{B}}O_{T}}$ is the normal vector to plane α passing through *O_α_*, the equation of plane α can be expressed as:

|  | $x_{T}\left(x-0.5x_{T}\right)+y_{T}\left(y-0.5y_{T}\right)+z_{T}\left(z-0.5z_{T}\right)=0$ | (21) |
| --- | --- | --- |

At this point, equation (4) can be substituted into equation (20) to obtain *θ_i,B_*:

|  | (22) |
| --- | --- |

And the result is:

|  | $A\sin\theta_{i,B}+B\cos\theta_{i,B}+C=0$ | (23) |
| --- | --- | --- |
|  |  | (24) |

Further solving equation (23) yields:

|  | $\left\{\begin{matrix} \sqrt{A^{2}+B^{2}}\sin\left(\theta_{i,B}+\beta\right)=-C \\ \beta=\arctan\frac{B}{A} \end{matrix}\right.$ | (25) |
| --- | --- | --- |
|  | $\theta_{i,B}=-\beta\pm\arcsin\left(\frac{C}{\sqrt{A^{2}+B^{2}}}\right)$ | (26) |

As indicated by equation (26), each result corresponds to two values with opposite signs, representing the two positions of *Q_i_* and *Q_i_*′ in **Figure S13** and **Figure S14**. Therefore, for a given pose and position of the moving platform, there are a total of eight possible combinations of motion configurations for the three branches.

**1.2.2 Kinematic analysis of ARS**

**1.2.2.1 Forward kinematics**

The ARSs can be equivalently modeled as the four-bar linkages, with the mechanical schematic shown in **Figure S16**. When Link 1, serving as the angular displacement input, undergoes an angular displacement *γ*_1_ about the *y_i_*-axis, Link 5 will correspondingly exhibit an angular displacement change of π-*γ*_5_, which is then transmitted to the branch chain of the passive rotation platform. Based on the geometric relationships, the kinematic equations for this mechanism can be established as follows:

|  | $\left\{\begin{matrix} l_{1}\sin\gamma_{1}+l_{2}\sin\gamma_{2}+l_{3}\sin\gamma_{3}+l_{4}\sin\gamma_{4}=l_{5}\sin\gamma_{5}+l_{6}\sin\gamma_{6}+l_{7} \\ l_{1}\cos\gamma_{1}+l_{2}\cos\gamma_{2}+l_{3}\cos\gamma_{3}+l_{4}\cos\gamma_{4}+l_{5}\cos\gamma_{5}+l_{6}\cos\gamma_{6}=l_{1}+l_{5} \\ \gamma_{4}=\gamma_{6} \\ \begin{matrix} \gamma_{2}=\gamma_{1}+\frac{\text{π}}{2} \\ \gamma_{5}=\gamma_{6}+\frac{\text{π}}{2} \end{matrix} \end{matrix}\right.$ | (27) |
| --- | --- | --- |


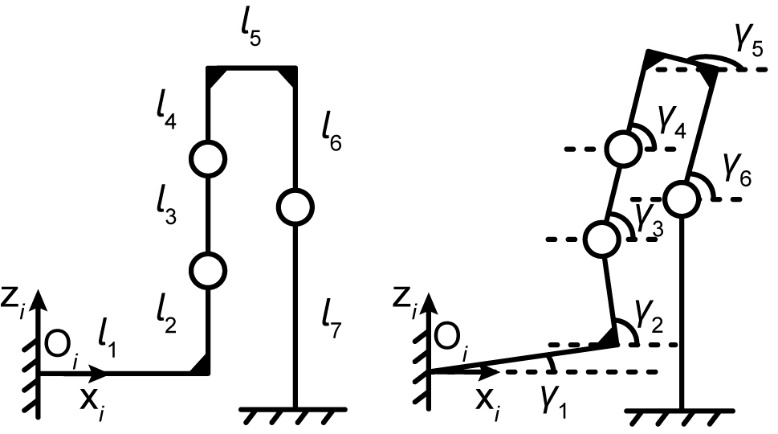


**Figure S16. Kinematic model of the ARS.**

When the input angular displacement *γ*_1_ is specified in the system of equations (27), the system yields a unique output of π-*γ*_5_.

**1.2.2.2 Inverse kinematics**

Similarly, since the system has a single degree of freedom, the input angular displacement *γ*_1_ can be solved using the system of equations (26) when the output angular displacement π-*γ*_5_ is specified.

**1.3 Kinematic and statics modeling based on the rigid-flexible coupling model of MOM**

This section establishes a rigid-flexible composite kinematic and mechanical model of the system by replacing the flexible hinges with equivalent components using the PRB method. The MOM contains a total of 30 flexible hinge joints. If moment equilibrium equations are solved for the PRB links in each hinge, the resulting computational complexity would make the rigid-flexible composite kinematic and static model exceedingly cumbersome. To simplify the mathematical model, each flexible hinge in the ARS is equivalently represented by four PRB links. In contrast, due to their relatively short length compared to the rigid links, the flexible hinges in the PRS are still treated as torsional springs with constant stiffness.

According to the principle of virtual work, when the system is in static equilibrium under external forces, the product of the virtual displacement at the output end *δ****W*** and the total external force ***Q****_tot_* equals the sum of the products of the virtual displacements and the corresponding resisting forces/moments at all internal joints. Specifically, for the parallel mechanism studied in this chapter, where all joints are revolute hinges, this relationship can be expressed as:

|  | $\mathbf{Q}_{tot}^{\text{T}}\delta\mathbf{W}=\mathbf{M}^{\text{T}}\delta\mathbf{\theta}$ | (28) |
| --- | --- | --- |

where $\mathbf{M}=\left[\begin{matrix} M_{1} & M_{2} & ... & M_{n} \end{matrix}\right]^{\text{T}}$ and $\delta\mathbf{\theta}=\left[\begin{matrix} \delta\theta_{1} & \delta\theta_{2} & ... & \delta\theta_{n} \end{matrix}\right]^{\text{T}}$ are the resisting torque generated by *n* hinges and the corresponding virtual angular displacement at the joints.

**1.3.1. Static analysis model for an individual branch of the PRS**

Based on the structural characteristics of the PRS, a mechanical modeling analysis is first performed for a single branch chain. Let the branch force borne by a single branch under the external force ***Q****_tot_* be denoted as ***Q****_i_*, and the resisting moment generated by each hinge in the branch be represented as $\mathbf{M}_{i}=\left[\begin{matrix} M_{i,1} & M_{i,2} & ... & M_{i,j} & ... & M_{i,m} \end{matrix}\right]^{\text{T}}\left(j=1,2,...,m\right)$. Here, *i* is the branch index, *j* is the hinge index within branch i, and m is the total number of hinges in branch *i*. Accordingly, equation (28) can be rewritten as the equilibrium equation for branch i:

|  | $\mathbf{Q}_{i}^{\text{T}}\delta\mathbf{W}_{i}=\mathbf{M}_{i}^{\text{T}}\delta\mathbf{\theta}_{i}$ | (29) |
| --- | --- | --- |

In the equation, $\delta\mathbf{\theta}_{i}=\left[\begin{matrix} \delta\theta_{i,1} & \delta\theta_{i,2} & ... & \delta\theta_{i,j} & ... & \delta\theta_{i,m} \end{matrix}\right]^{\text{T}}\left(j=1,2,...,m\right)$ represents the virtual angular displacement of each hinge in branch *i*. Since ***Q****_i_* and ***M****_i_* depend on the form of the external load and the material/geometric properties of the flexible hinges, respectively, solving equation (29) only requires clarifying the mathematical relationship between the virtual displacement at the branch output and the virtual displacements of the individual hinges. According to the kinematics theory of serial mechanisms:

|  | $\delta\mathbf{W}_{i}=\mathbf{J}_{i}\delta\mathbf{\theta}_{i}$ | (30) |
| --- | --- | --- |

Moreover, the motion screw system of the mechanism is the geometric entity represented by the velocity Jacobian matrix, ***J****_i_*. Therefore, equation (30) can be rewritten as:

|  | $\delta\mathbf{W}_{i}=\mathbf{\$}_{i}\delta\mathbf{\theta}_{i}$ | (31) |
| --- | --- | --- |

In the equation, represents the motion screw system of branch *i*. The following analyzes the motion screw system of branch *i*:

The common intersection point *Q_i_* is selected as the origin of the coordinate system. The direction of the motion screw ***$****_i_*_,2_ is taken as the *y_i_*-axis, while the *x_i_*-axis is defined as the line perpendicular to ***$****_i_*_,2_ and lying in the plane formed by ***$****_i_*_,2_ and ***$****_i_*_,5_. The *z_i_*-axis is determined by the right-hand rule as vertically upward, as shown in **Figure S17**.


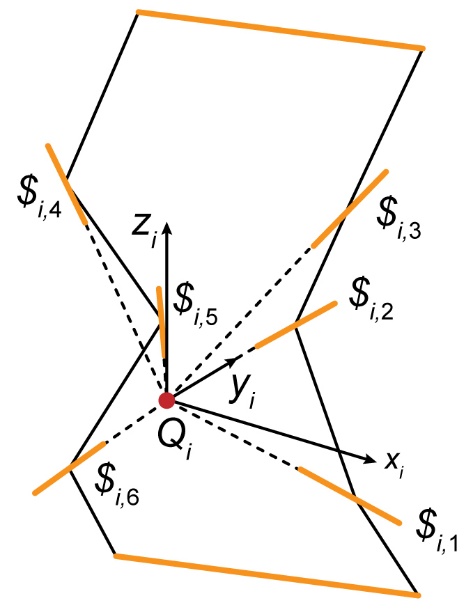


**Figure S17.** **Analysis of the Motion Screw in a Single Branch.**

According to screw theory, the motion screw system in a single right branch chain, comprising three revolute joints, is given as follows:

|  |  | (32) |
| --- | --- | --- |

The reciprocal screw system is then solved. Let the restrict screw be denoted as $\mathbf{w}=\left(\omega_{\omega};v_{\omega}\right)$, then the restrict screw system forms the solution space of the following system of linear equations:

|  | $\left\{\begin{matrix} x_{1}v_{wx}+y_{1}v_{wy}+z_{1}v_{wz}=0 \\ y_{2}v_{wy}=0 \\ x_{3}v_{wx}+y_{3}v_{wy}+z_{3}v_{wz}=0 \end{matrix}\right.$ | (33) |
| --- | --- | --- |

Its matrix form is:

|  | $\underbrace{\left(\begin{matrix} x_{1} & y_{1} & z_{1} \\ 0 & y_{2} & 0 \\ x_{3} & y_{3} & z_{3} \end{matrix}\right)}_{M}\left(\begin{matrix} v_{wx} \\ v_{wy} \\ v_{wz} \end{matrix}\right)=0$ | (34) |
| --- | --- | --- |

Under the condition that the coefficient matrix ***M*** is full-rank, meaning r***_M_*** = rank(***M***) = 3, the dimension of the restrict screw system is then:

|  | $\dim=3+\left(3-r_{\mathbf{M}}\right)=6-r_{\mathbf{M}}=3$ | (35) |
| --- | --- | --- |

Since $\omega_{1}$, $\omega_{2}$, and $\omega_{3}$ contained in $\omega_{\omega}$ are linearly independent, the reciprocal screw system of the mechanism can be expressed as:

|  |  | (36) |
| --- | --- | --- |

Similarly, the motion screw system and the reciprocal screw system for the left branch chain are calculated as follows:

|  | $\left\{\begin{matrix} \mathbf{\$}_{i\text{,4}}=\left(\begin{matrix} x_{4} & y_{4} & z_{4} & \text{;} & 0 & 0 & 0 \end{matrix}\right) \\ \mathbf{\$}_{i\text{,5}}=\left(\begin{matrix} x_{5} & y_{5} & 0 & \text{;} & 0 & 0 & 0 \end{matrix}\right) \\ \mathbf{\$}_{i\text{,6}}=\left(\begin{matrix} x_{6} & y_{6} & z_{6} & \text{;} & 0 & 0 & 0 \end{matrix}\right) \end{matrix}\right.$ | (37) |
| --- | --- | --- |
|  |  | (38) |

It can be observed that the left and right branch chains share an identical representation of the reciprocal screw system, demonstrating that the three pairs of revolute joints impose three common constraints. Hence, the reciprocal screw system of the folding branch containing six revolute joints can be simplified as:

|  |  | (39) |
| --- | --- | --- |

An analysis of the reciprocal screw system of the branch chain reveals that it only permits the moment about any axis while constraining input forces in all directions. This indicates that the folding branch chain, comprising six revolute joints, possesses three rotational degrees of freedom. That is, its topological configuration can be simplified to either a series of three revolute pairs or a spherical joint. Accordingly, the motion screw system of a single branch chain containing eight revolute joints can be established as follows:

|  |  | (40) |
| --- | --- | --- |

According to the expression given in equation (31), the virtual angular displacement of the hinge still involves the inverse matrix of the motion screw system. To simplify the calculation procedure, a force screw ***R****_i,j_* is introduced, whose physical meaning is the generalized force vector causing motion at joint *j*. Therefore, the reciprocal product between ***R****_i,j_* and all motion screws in branch *i*, except ***$****_i,j_*, is **0**. Hence,

|  | $\mathbf{R}_{i}^{\text{T}}\delta\mathbf{W}_{i}=\mathbf{R}_{i}^{\text{T}}\mathbf{\$}_{i}\delta\mathbf{\theta}_{i}$ | (41) |
| --- | --- | --- |
|  | $\mathbf{R}_{i}^{\text{T}}\delta\mathbf{W}_{i}=\left[\begin{matrix} \mathbf{R}_{i,1}^{\text{T}}\mathbf{\$}_{i,1} & & & \\ & \mathbf{R}_{i,2}^{\text{T}}\mathbf{\$}_{i,2} & & \\ & & ... & \\ & & & \mathbf{R}_{i,m}^{\text{T}}\mathbf{\$}_{i,m} \end{matrix}\right]\delta\mathbf{\theta}_{i}$ | (42) |
|  | $\delta\mathbf{\theta}_{i}=\left[\begin{matrix} \frac{1}{\mathbf{R}_{i,1}^{\text{T}}\mathbf{\$}_{i,1}} & & & \\ & \frac{1}{\mathbf{R}_{i,2}^{\text{T}}\mathbf{\$}_{i,2}} & & \\ & & ... & \\ & & & \frac{1}{\mathbf{R}_{i,m}^{\text{T}}\mathbf{\$}_{i,m}} \end{matrix}\right]\mathbf{R}_{i}^{\text{T}}\delta\mathbf{W}_{i}$ | (43) |

Here, $\mathbf{R}_{i}=\left[\begin{matrix} \mathbf{R}_{i,1}^{\text{T}} & \mathbf{R}_{i,2}^{\text{T}} & ... & \mathbf{R}_{i,j}^{\text{T}} & ... & \mathbf{R}_{i,m}^{\text{T}} \end{matrix}\right]$. Substituting equation (43) into equation (29) yields:

|  | $\mathbf{Q}_{i}^{\text{T}}=\mathbf{M}_{i}^{\text{T}}\left[\begin{matrix} \frac{1}{\mathbf{R}_{i,1}^{\text{T}}\mathbf{\$}_{i,1}} & & & \\ & \frac{1}{\mathbf{R}_{i,2}^{\text{T}}\mathbf{\$}_{i,2}} & & \\ & & ... & \\ & & & \frac{1}{\mathbf{R}_{i,m}^{\text{T}}\mathbf{\$}_{i,m}} \end{matrix}\right]\mathbf{R}_{\mathbf{i}}^{\text{T}}$ | | | (44) |
| --- | --- | --- | --- | --- |
|  | | $\mathbf{Q}_{i}=\sum_{j=1}^{m} \frac{\mathbf{R}_{i,j}\mathbf{M}_{i,j}}{\mathbf{R}_{i,j}^{\text{T}}\mathbf{\$}_{i,j}}$ | (45) | |

Based on the motion screw system of a single branch chain given in equation (40), the corresponding force screw system ***R****_i_* can be obtained as follows:

| $\left\{\begin{matrix} \mathbf{R}_{1,T}^{\text{T}}=\left[\begin{matrix} \left(cy-bz\right)\left(bx-ay\right) & \left(az-cx\right)\left(bx-ay\right) & P^{2} & ; & 0 & 0 & 0 \end{matrix}\right]^{\text{T}} \\ \mathbf{R}_{1,p1}^{\text{T}}=\left[\begin{matrix} 0 & 0 & -a & ; & bx-ay & 0 & 0 \end{matrix}\right]^{\text{T}} \\ \mathbf{R}_{1,p2}^{\text{T}}=\left[\begin{matrix} 0 & 0 & -b & ; & 0 & bx-ay & 0 \end{matrix}\right]^{\text{T}} \\ \mathbf{R}_{1,p3}^{\text{T}}=\left[\begin{matrix} -\frac{c\left(cy-bz\right)}{P} & -\frac{c\left(az-cx\right)}{P} & 0 & ; & 0 & 0 & P \end{matrix}\right]^{\text{T}} \\ \mathbf{R}_{1,B}^{\text{T}}=\left[\begin{matrix} \left(bz-cy\right)\left(bx-ay\right) & \left(cx-az\right)\left(bx-ay\right) & P^{2} & ; & 0 & 0 & 0 \end{matrix}\right]^{\text{T}} \end{matrix}\right.$ | (46) |
| --- | --- |

Here, $P=\sqrt{\left(cy-bz\right)^{2}+\left(az-cx\right)^{2}}$. At this point, a mathematical model has been established relating the external load applied to a single branch chain to the internal deformation and reaction forces of its hinges. Since the motion screw system in equation (40) is defined in the local coordinate system *Q_i_-x_i_y_i_z_i_*, it must be transformed into the global coordinate system O_B_-XYZ using a coordinate transformation matrix.

In spatial mechanics, the screw transformation between two coordinate systems can be concisely represented by an adjoint matrix. Let the rigid body transformation from the coordinate system O_B_-XYZ to *Q_i_-x_i_y_i_z_i_* be described by a translation vector ***T****_Bi_* and a rotation matrix ***R****_Bi_*. Then:

|  |  | | | | | (47) |
| --- | --- | --- | --- | --- | --- | --- |
|  | |  | | | (48) | |
|  | | | | $\mathbf{\$}=\mathbf{Ad}_{Bi}\mathbf{\$}'$ | (49) | | |
|  | | | | $\mathbf{Ad}_{Bi}=\left[\begin{matrix} \mathbf{R}_{Bi} & 0 \\ \mathbf{T}_{Bi}\mathbf{R}_{Bi} & \mathbf{R}_{Bi} \end{matrix}\right]$ | (50) | | |

In this expression, ***$*** represents the screw quantity expressed in the local coordinate system *Q_i_-x_i_y_i_z_i_*, while ***$***′ denotes its representation in the global coordinate system O_B_-XYZ. In equation (48), ***R****_Z,i_* (*φ_Z,i_*), ***R****_Z,i_* (*κ*), and ***R****_Z,i_* (*υ*) are the rotation matrices about the Z-axis, and ***R****_Y,i_* (*ψ*) is the rotation matrix about the Y-axis. Here, *φ_Z,i_* and *ψ* can be obtained from **Table. S2** and equation (16), respectively, and κ is the angle between the Y-axis and the vector resulting from the cross product of the Z-axis and :

|  | $\kappa=\arctan\left(-b/a\right)$ | (51) |
| --- | --- | --- |

*υ* is the angle between the *y_i_*-axes of the coordinate systems *B_i,o_-x_i_y_i_z_i_* and *Q_i_-x_i_y_i_z_i_*. The calculation method is illustrated in **Figure S18**: after determining the position coordinates of point *E_B_* on the fixed platform (determined by structural design parameters) in the global coordinate system, forward kinematics is used to calculate the position coordinates of its symmetric point *E_T_* on the moving platform with respect to the mirror plane α. Subsequently, the length *h_i_* of the vector and the position coordinates of its perpendicular foot E_α_ on the mirror plane α are solved. Then, utilizing the projection angle *β_i_* and the vector angle *ς_i_*, the direction vector of the screw ***$****_i_*_,2_ (y_i_-axis) is obtained. Finally, *υ* is solved using the angle formula:

|  | $\upsilon=\arccos\left(\frac{\overrightarrow{B_{i,O}E_{B}}\cdot\omega_{i,2}}{\left\Vert\overrightarrow{B_{i,O}E_{B}}\right\Vert\cdot\left\Vert\omega_{i,2}\right\Vert}\right)$ | (52) |
| --- | --- | --- |


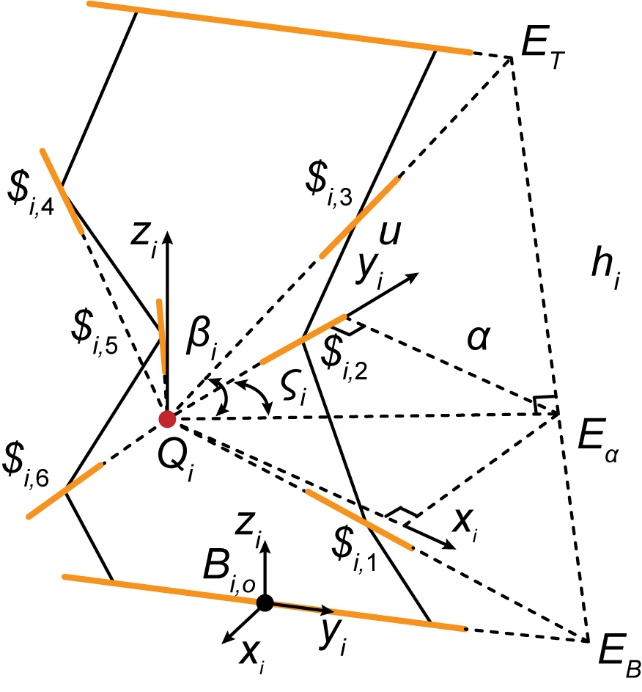


**Figure S18.** **The geometric relationship in a single branch chain of PRS during statics modeling.**

Since the positional coordinates of points *E_T_*, *E_B_*, *E_α_*, and *Q_i_* in **Figure S18** can be determined from the geometric relationships described above, it is possible to construct the normal vector directions of the three right-side folding surfaces represented by ***$****_i_*_,1_-***$****_i_*_,2_-***$****_i_*_,3_ within a single branch chain. Subsequently, the angles *θ_i,j_* between these folding surfaces can be solved. Note that the same method can be applied to determine the angles of the three left-side folding surfaces. At this stage,

|  | $M_{i,j}=K_{\theta}W_{i,j}\theta_{i,j}$ | (53) |
| --- | --- | --- |

where *K_θ_* is the equivalent torsional stiffness of the hinge and *W_i,j_* is the width of the hinge. At this point, all parameters on the right-hand side of equation (45) have been expressed in analytical form.

**1.3.2 Static analysis model for the ARSs**

This section models the flexible hinges in the ARSs using the PRB model and conducts a static analysis of the ARSs by applying moment equilibrium equations. A detailed description of the PRB model can be found in the relevant literature.

For any flexible hinge Joint*_n_* (*n*=*a*, *b*, *c*), a local coordinate system *O_n_-X_n_Z_n_* is established at its leading-end link, as shown in **Figure S19.** The force analysis for each flexible segment containing three PRB links is as follows:

|  | $\left\{\begin{matrix} M_{i,n}=K_{\gamma_{1}}\gamma_{m,1}+F_{oz,n}^{(n)}l_{n,1} \\ K_{\gamma_{1}}\gamma_{m,1}=K_{\gamma_{2}}\gamma_{m,2}+F_{oz,n}^{(n)}l_{n,2}\cos\gamma_{m,1}-F_{ox,n}^{(n)}l_{n,2}\sin\gamma_{m,1} \\ K_{\gamma_{2}}\gamma_{m,2}=K_{\gamma_{3}}\gamma_{m,3}+F_{oz,n}^{(n)}l_{n,3}\cos\left(\gamma_{m,1}+\gamma_{m,2}\right)-F_{ox,n}^{(n)}l_{n,3}\sin\left(\gamma_{m,1}+\gamma_{m,2}\right) \\ K_{\gamma_{3}}\gamma_{m,3}=F_{oz,n}^{(n)}l_{n,4}\cos\left(\gamma_{m,1}+\gamma_{m,2}+\gamma_{m,3}\right)-F_{ox,n}^{(n)}l_{n,4}\sin\left(\gamma_{m,1}+\gamma_{m,2}+\gamma_{m,3}\right)+M_{o,n} \\ F_{ix,n}^{(n)}=F_{ox,n}^{(n)} \\ F_{iz,n}^{(n)}=F_{oz,n}^{(n)} \end{matrix}\right.$ | | | (54) | |
| --- | --- | --- | --- | --- | --- |
|  | | $\left\{\begin{matrix} \gamma_{m+1}=\gamma_{m}-\sum_{k=1}^{3} \gamma_{m,k}(m=\text{2,3}) \\ \gamma_{m-1}=\gamma_{m}-\sum_{k=1}^{3} \gamma_{m,k}(m=7) \end{matrix}\right.$ | (55) | |

Where $F_{ix,n}^{(n)}$, $F_{iy,n}^{(n)}$, and $M_{i,n}^{(n)}$ represent the external forces and moments applied to the connection points between the input link and the PRB link, respectively. And $F_{ox,n}^{(n)}$, $F_{oy,n}^{(n)}$, and $M_{o,n}^{(n)}$ represent the external forces and moments applied to the connection points between the input link and the PRB link, respectively. The subscript n denotes the joint number, while the superscript (*n*) denotes the local coordinate system *O_n_-X_n_Z_n_*. m indicates the serial number of the input rigid link in each flexure hinge system in the global system, as shown in **Figure S19**. The equation (54) set has a total of 11 unknowns, including 4 forces, 2 moments, 2 global rotation angles, and 3 subsystem rotation angles.

Equation (54) can be simplified to obtain:

|  | $\left\{\begin{matrix} F_{ox,n}^{(n)}=F_{ix,n}^{(n)} \\ F_{oz,n}^{(n)}=F_{iz,n}^{(n)} \\ M_{o,n}=M_{i,n}+\left[\begin{matrix} F_{ix,n}^{(n)} & -F_{iz,n}^{(n)} \end{matrix}\right]\left[\begin{matrix} \mathbf{l} & 0 \\ 0 & \mathbf{l} \end{matrix}\right]\left[\begin{matrix} \sin\mathbf{\gamma} \\ \cos\mathbf{\gamma} \end{matrix}\right] \end{matrix}\right.$ | (56) |
| --- | --- | --- |

where，

|  | | $\mathbf{l}=\left[l_{n,1},l_{n,2},l_{n,3},l_{n,4}\right]$ | (57) | |
| --- | --- | --- | --- | --- |
|  | $\mathbf{\gamma}=\left[0,\gamma_{m,1},\gamma_{m,1}+\gamma_{m,2},\gamma_{m,1}+\gamma_{m,2}+\gamma_{m,3}\right]^{\text{T}}$ | | | (58) |

Therefore, when the values of 4 out of the 11 unknowns are known, such as the force $F_{ix,n}^{(n)}$, $F_{iz,n}^{(n)}$, the moment $M_{i,n}$ at the input link, and its rotation angle $\gamma_{m}$ in the global coordinate system, the remaining variables can be solved. These include the output parameters corresponding to the input conditions: the force $F_{o,n}^{(n)}$ and moment $M_{o,n}$ at the output link, its rotation angle $\gamma_{m+1}$, as well as the internal sub-angles $\gamma_{m,i}$.

Focusing on the system level, for the connecting link $l_{5}$, based on the force analysis of the fixed platform of the PRS, it is subjected to a load moment $M_{load}$ about the Y-axis. Therefore, for the connecting link $l_{5}$, the following condition is satisfied:

|  | $\left\{\begin{matrix} M_{o,b}+M_{load}+M_{o,c}=0 \\ F_{ox,b}+F_{ox,c}=0 \\ F_{oz,b}+F_{oz,c}=0 \end{matrix}\right.$ | (59) |
| --- | --- | --- |

According to the kinematic relationship, it can be obtained that:

| $\left\{\begin{matrix} \begin{matrix} l_{1}\cos\gamma_{1}+l_{2}\cos\gamma_{2}+l_{3}\cos\gamma_{3}+l_{4}\cos\gamma_{4}+\sum_{k=1}^{k=4} l_{a,k}\cos(\gamma_{2}-\sum_{j=0}^{j=k-1} \gamma_{2,j})+\sum_{k=1}^{k=4} l_{b,k}\cos(\gamma_{3}-\sum_{j=0}^{j=k-1} \gamma_{3,j})= \\ l_{0}+l_{5}\cos\gamma_{5}+l_{6}\cos\gamma_{6}+\sum_{k=1}^{k=4} l_{c,k}\cos(\frac{\pi}{2}-\sum_{j=0}^{j=k-1} \gamma_{7,j}) \end{matrix} \\ \begin{matrix} l_{1}\sin\gamma_{1}+l_{2}\sin\gamma_{2}+l_{3}\sin\gamma_{3}++l_{4}\sin\gamma_{4}+\sum_{k=1}^{k=4} l_{a,k}\sin(\gamma_{2}-\sum_{j=0}^{j=k-1} \gamma_{2,j})+\sum_{k=1}^{k=4} l_{b,k}\sin(\gamma_{3}-\sum_{j=0}^{j=k-1} \gamma_{3,j})= \\ l_{5}\sin\gamma_{5}+l_{6}\sin\gamma_{6}+l_{7}+\sum_{k=1}^{k=4} l_{c,k}\sin(\frac{\pi}{2}-\sum_{j=0}^{j=k-1} \gamma_{7,j}) \end{matrix} \end{matrix}\right.$ | (60) |
| --- | --- |

where $\gamma_{m,0}=0$. Consequently, when performing a forward solution of the equation system for the three flexible hinge mechanisms, there are 8 independent variables ($F_{Lx}$, $F_{Lz}$, $M_{L}$, $\gamma_{1}/\gamma_{5}$, $F_{Rx}$, $F_{Rz}$, $M_{R}$, *M_load_*). Equations (59) and (60) provide a total of 5 equations. Additionally, for the connecting link $l_{5}$, the output link rotation angles in the left and right flexible hinge systems are identical, i.e.,

|  | $\gamma_{5}=\gamma_{3}-\sum_{k=1}^{3} \gamma_{3,k}+\frac{\text{π}}{2}=\text{π}-\sum_{k=1}^{3} \gamma_{7,k}$ | (61) |
| --- | --- | --- |


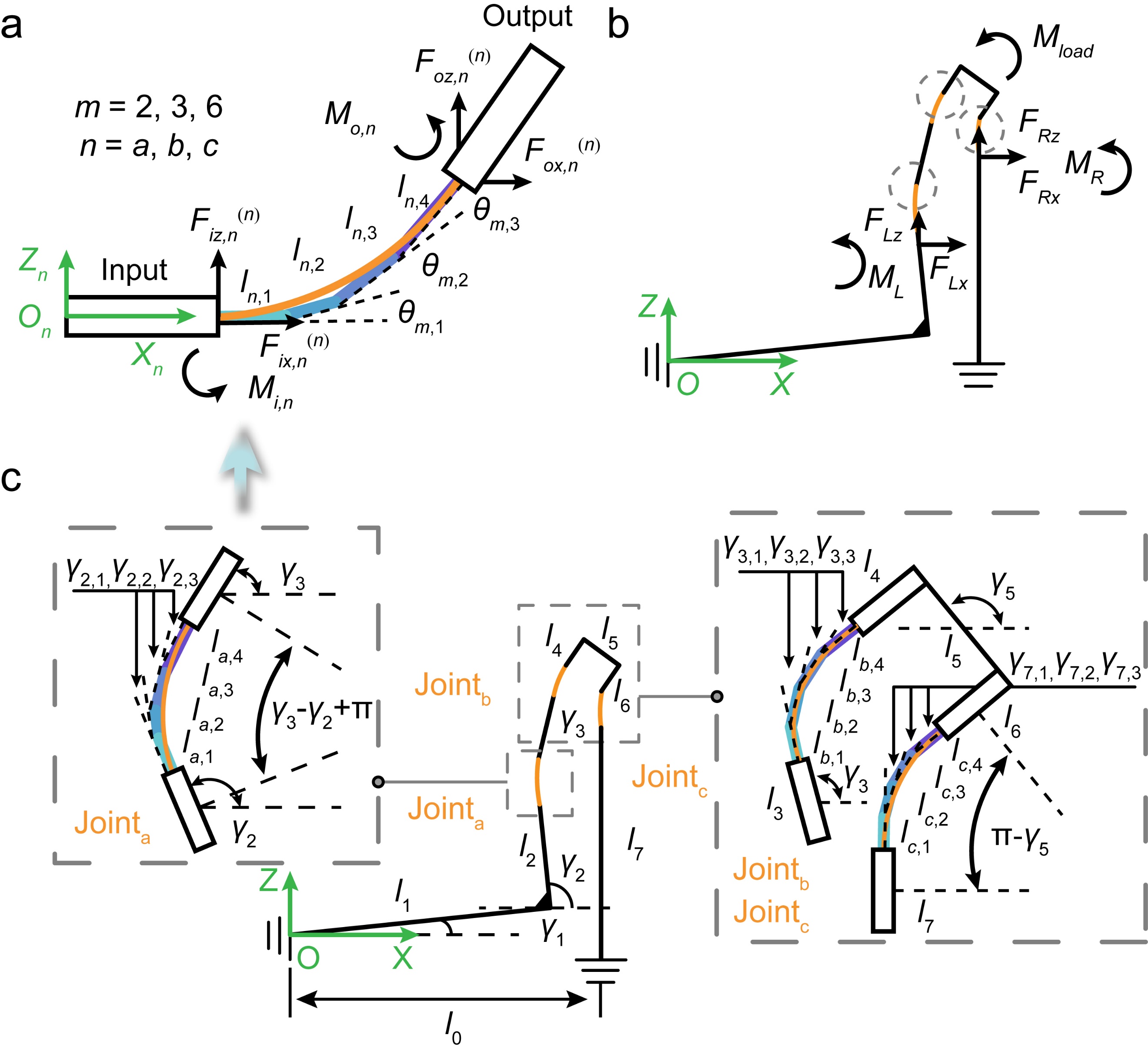


**Figure S19.** **Static Analysis of the ARSs using the PRB Model.** **a,** Deformation of the fundamental unit; **b,** Overall force distribution; **c,** Static analysis of the ARS with three flexible hinges.

Therefore, when the input rotation angle and the input actuation force are specified (with $\gamma_{1}$ and $F_{Lz}$ known), the static and kinematic states of the above mechanism yield a unique solution (i.e., the output angle and output torque, corresponding to the forward solution of the system). Conversely, when the external load configuration and the output rotation angle are given (with *M_load_* and $\gamma_{5}$ known), the inverse relationship of the system (i.e., the input actuation force and input angle) can also be determined.

**1.3.3 Kinematic and statics modeling of the complete system**

After completing the rigid-flexible composite kinematic and static modeling of the PRS and ARSs, it is necessary to construct a comprehensive kinematic and static model of the integrated system using coordinate transformation matrices and geometric relationships. **Figure S20** shows the mechanism schematic of a single branch chain of the complete motion platform. The connecting link 1 in the PRS is fixed to Joint*_c_* of the ARS via an angular adapter. To simplify the analysis, the rotation center of connecting link 1 is considered to coincide with Joint*_c_*, and the coordinate system *B_i,o_-x_i_y_i_z_i_* is established as shown in the figure.


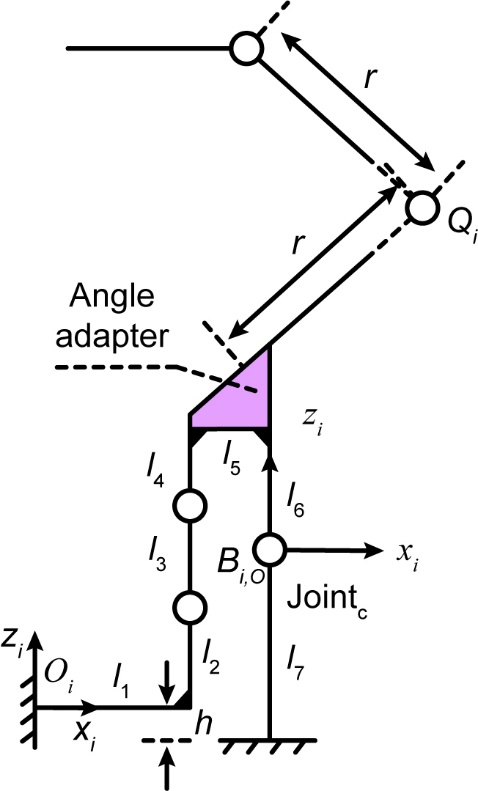


**Figure S20.** **Schematic Diagram of a Single Branch Mechanism in the Complete System**

The rigid body transformation from the coordinate system O_B_-XYZ to *O_i_-x_i_y_i_z_i_* is described by the translation vector ***T****_Oi_*:

|  | $\mathbf{T}_{Oi}=\mathbf{T}_{XZ,i}\mathbf{T}_{Bi}$ | (62) |
| --- | --- | --- |

where，

|  | $\mathbf{T}_{XZ,i}=\left[\begin{matrix} 1 & 0 & 0 & -l_{1}-l_{5} \\ 0 & 1 & 0 & 0 \\ 0 & 0 & 1 & h-l_{7} \\ 0 & 0 & 1 & 1 \end{matrix}\right]$ | (63) |
| --- | --- | --- |

At this stage, the kinematic and static model for a single branch of the parallel motion platform has been fully established. Consequently, the equilibrium equation for the total reaction force of the platform, which comprises three kinematic branches, can be expressed as:

|  | $\mathbf{Q}_{tol}=\sum_{i=1}^{3} \sum_{j=1}^{m} \frac{\mathbf{R}_{i,j}\mathbf{M}_{i,j}}{\mathbf{R}_{i,j}^{\text{T}}\mathbf{\$}_{i,j}}$ | (64) |
| --- | --- | --- |

By using equation (64), the general procedure for inverse statics/kinematics analysis of the system can be defined as follows:

Given the center position coordinates *O_T_ (x_T_，y_T_，z_T_)* of the PRS platform under the load ***Q****_tol_*, the input angles *θ_i,B_* and the required torque *M_i,B_* of the three motion branches of the PRS are solved. These values are then substituted into the moment equilibrium equations of the ARSs to finally determine the drive displacement (*^i^*)*γ*_1_ and drive force (*^i^*)*F_Lz_* of the actuator, where the superscript (*i*) denotes the branch index. The procedure for forward statics/kinematics analysis follows a similar logic.

**1.3.4 Kinematic and mechanical performance evaluation of the system**

**1.3.4.1 Range of motion**

After obtaining the driving force and displacement of the PZT actuators, these values are used as inputs to evaluate the output displacement of the MOM through the forward kinematics solution strategy. The evaluation includes both single DOF linear displacement and angular displacement.

**1.3.4.2 Static stiffness**

Based on the established theoretical model, the static stiffness analysis for the system can be developed. The MOM is characterized by two key stiffness performance indicators: axial stiffness *K_L_*​ and steering stiffness *K_θ_*, as illustrated in **Figure S21**.


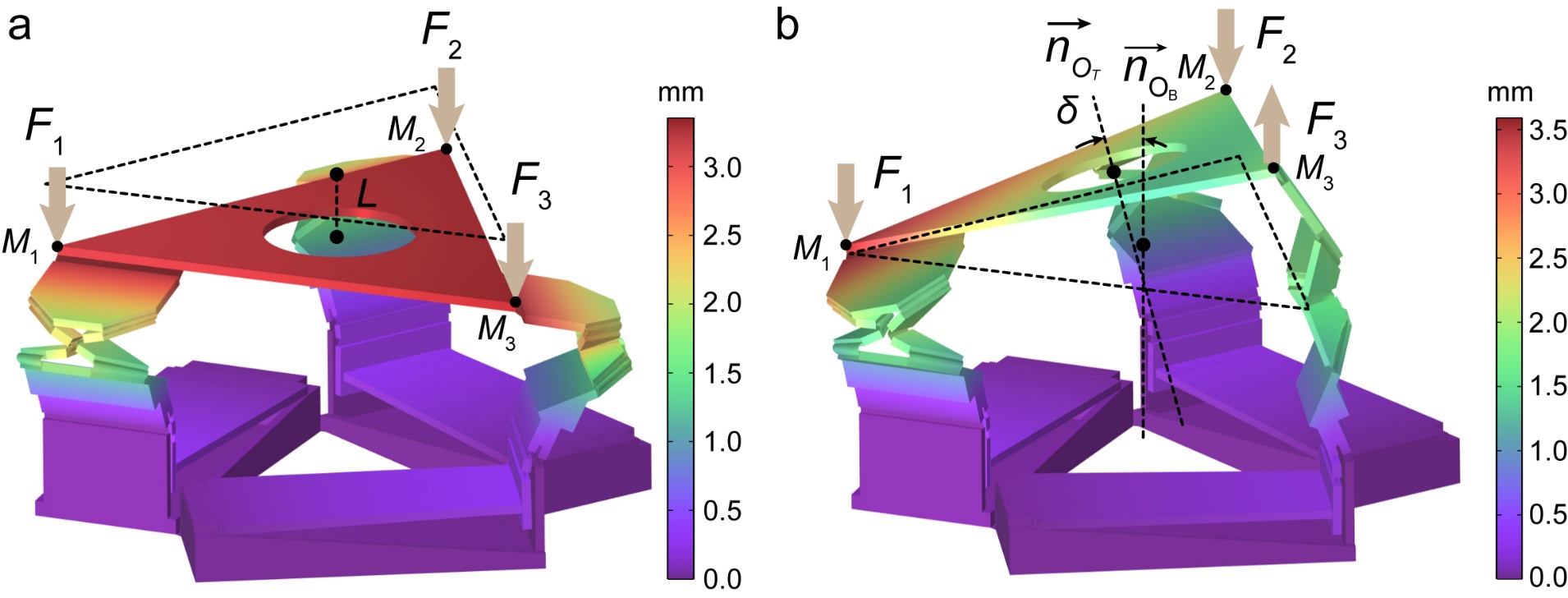


**Figure S21. Stiffness Evaluation of the MOM. a,** Axial stiffness evaluation; **b,** Steering stiffness evaluation.

a) Axial stiffness *K_L_* evaluation

When the moving platform of the system undergoes a linear displacement *L* along the Z-axis under an external load ***Q****_tol_*, the parameters *θ_i,j_*, *θ_i,T_*, and *θ_i,B_* can be determined using the inverse kinematics of the PRS, as illustrated in **Figure S21a**. Subsequently, based on the input driving force and displacement of the PZT actuators, *M_i,B_* can be solved. Finally, the analytical expression of ***Q****_tol_* is derived through the forward kinematics of the PRS.

|  | ${}^{\left(i\right)}F_{Lz}=F_{b,ext}$ | (65) |
| --- | --- | --- |
|  | ${}^{\left(i\right)}\gamma_{1}=0$ | (66) |
|  | $K_{L}=\frac{\left\vert\mathbf{Q}_{tol}\right\vert}{L}$ | (67) |

b) Steering stiffness *K_θ_* evaluation

The solution strategy for the steering stiffness *K_θ_* is the same as that for the axial stiffness *K_L_*​, with the only additional requirement being to specify the moment arm ($\left|\overrightarrow{PO_{T}}\right|$) between the external load application point *M* and the center point *O_T_* of the moving platform:

|  | $K_{\theta}=\frac{\left\vert\mathbf{Q}_{tol}\right\vert\cdot\left\vert\overrightarrow{PO_{T}}\right\vert}{\delta}$ | (68) |
| --- | --- | --- |

**1.3.5 Influence of the hollowed dimension ratio γ on system performance**

For the motion branch chains, in addition to the dimensions of each rigid link and flexible hinge, the hollowed dimension ratio ($\gamma=\delta/r$) is equally critical in constraining their kinematic and mechanical performance. Therefore, utilizing the theoretical model established above, a preliminary evaluation is conducted on how *γ* affects the driving capability, and static stiffness of MOM. The dimensions and material properties of each link in the system are provided in **Table S3**.

**1.3.5.1 Driving ability**

By specifying the linear and angular displacements of the upper platform in the parallel motion system, the relationship between the actuation force ^(1^*)F_Lz_* of a single actuator and the hollow ratio *γ* was evaluated, as shown in **Figure S22**. The results clearly indicate that as the displacement increases, the required actuation force also increases. This is because larger end displacements lead to greater deformation of the flexible hinges within the system, and the resulting equivalent spring force reacts against the actuator. Furthermore, when the output displacement is fixed, the reaction force excited by the passive rotation platform gradually decreases with increasing *γ*, which also reduces the load demand on the actuator.

Therefore, after defining the output displacement requirements of the system, it is essential to evaluate the load capacity of the actuator within the given dimensional constraints. This ensures an optimal trade-off between the actuator capability and *γ*, leading to an optimized system design.


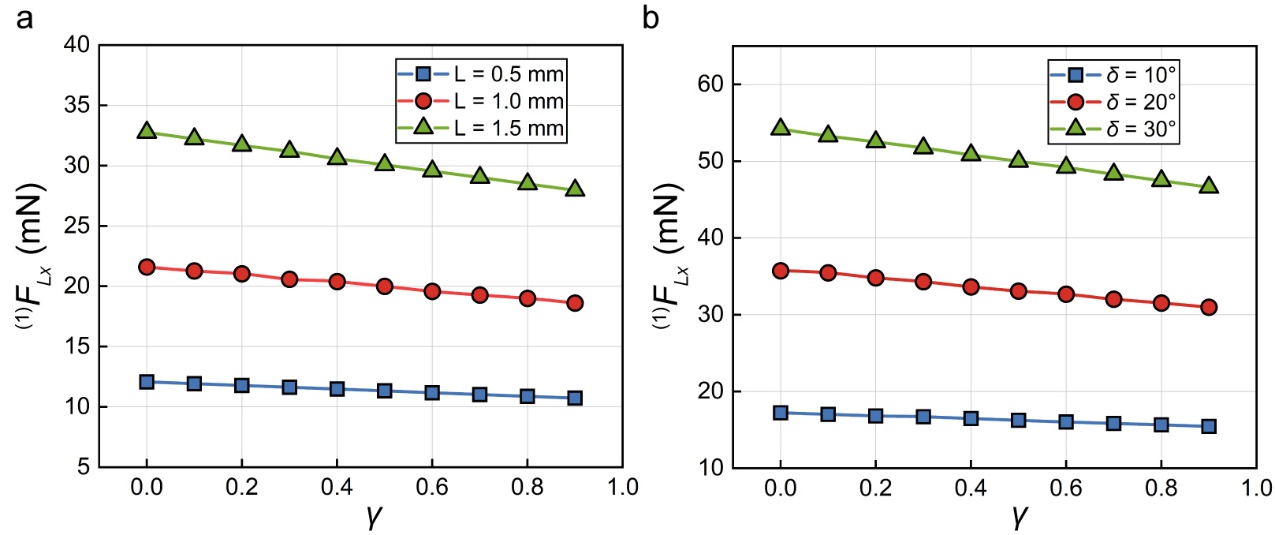


**Figure S22.** **Analysis for the driving capability of MOM. a,** Under specified linear displacement; **b,** Under specified angular displacement.

**1.3.5.2 Static stiffness**

The static stiffness of the system also depends on the selection of *γ*. For this purpose, following the example in **Figure S21**, different magnitudes of load forces were applied to the three vertices of the upper platform. Using the theoretical model described above, the axial stiffness and steering stiffness of the parallel rotation platform were evaluated, and a comparative analysis was performed with commercial finite element software, as shown in **Figure S23**.

The results demonstrate a trend consistent with expectations: as *γ* increases, both the axial and steering stiffness of the system gradually decrease. However, since the mechanical model developed in this study does not account for the nonlinearity in the equivalent stiffness of flexible hinges in the passive rotation platform under large-deflection deformation, the calculated results are lower than the simulation results. This discrepancy becomes more pronounced as the deformation of the hinges increases.


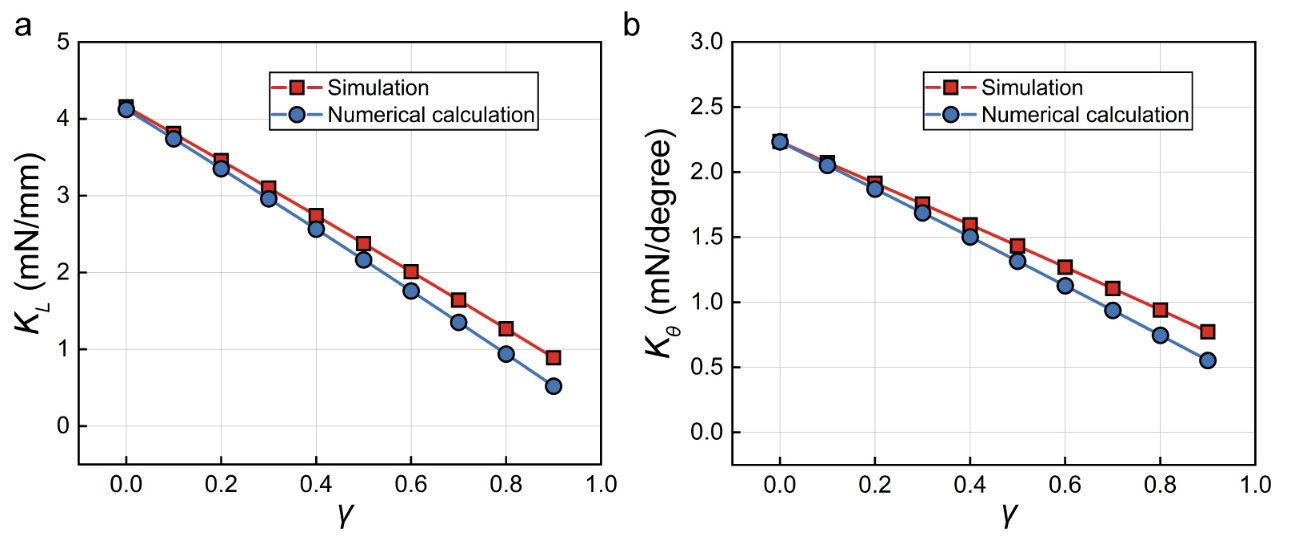


**Figure S23.** **Analysis for the static stiffness of MOM.** **a,** Axial stiffness; **b,** Steering stiffness.

**1.3.5.3 Resonant frequency and operational bandwidth of MOM in single DOF rotational motion**

To evaluate the dynamic performance of the MOM, a preliminary analysis of the resonant frequency and operational bandwidth of its single DOF rotational motion was conducted using finite element analysis software, as shown in **Figure S24**. It can be observed that when the output capability of the piezoelectric actuator is fixed, the output angle of the parallel motion platform increases with the increase of *γ*, while its operational bandwidth exhibits the opposite trend. Furthermore, **Figure S24b** indicates that when *γ* lies in the range of 0.4 to 0.8, the operational bandwidth is significantly influenced by *γ*. This is because when *γ* exceeds 0.8, the global stiffness of the system is primarily governed by the ARSs, whereas when *γ* falls below 0.4, the static stiffness of the PRS notably surpasses that of the ARSs. As a result, the variation in operational bandwidth remains relatively moderate within these two intervals. However, when the stiffnesses of the two components are comparable, changes in *γ* markedly affect the relative stiffness ($\Delta K_{\theta}/\Delta\gamma$) of the system, leading to a steeper slope in the curve within this intermediate range. This observation provides valuable insight for the selection of *γ* in the system design.


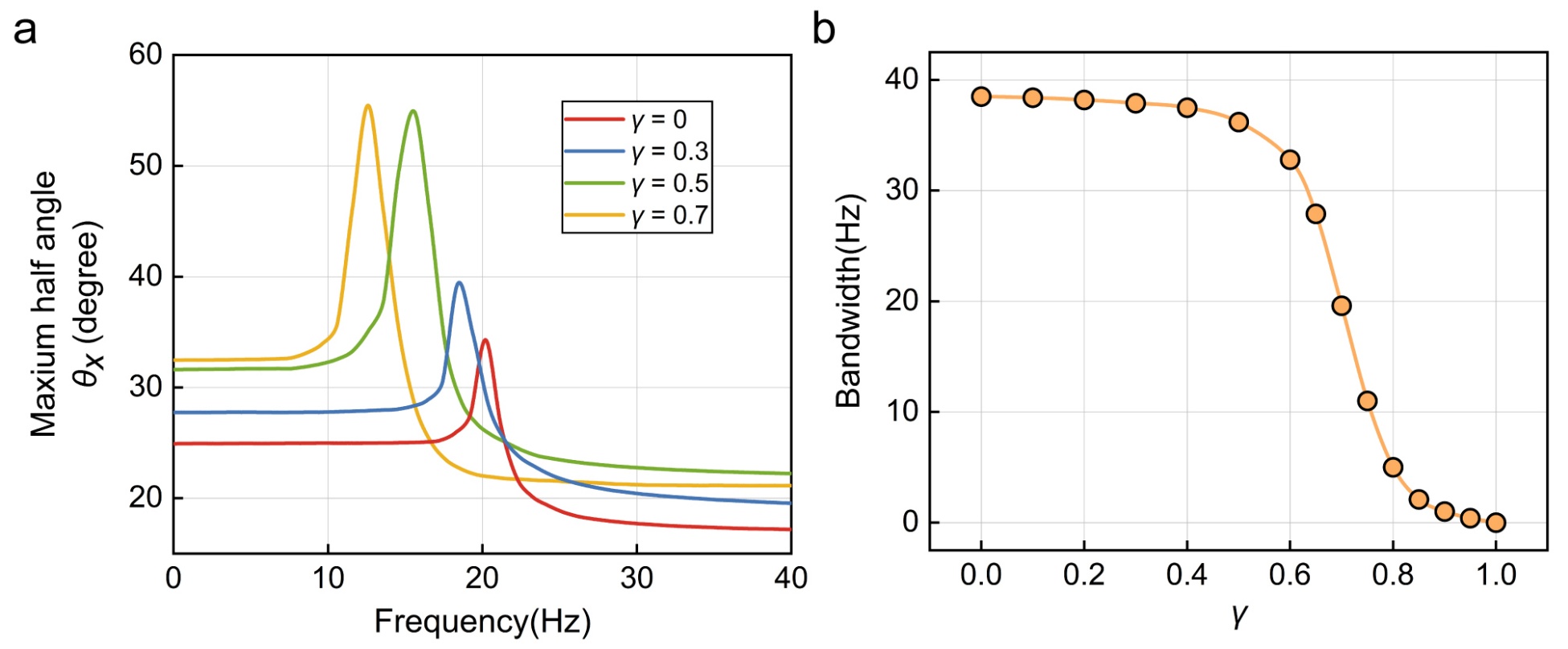


**Figure S24.** **Analysis of resonant frequency and operational bandwidth for the PRS under single DOF rotational motion. a,** Amplitude-frequency response characteristics under different *γ* values; **b,** Variation of the operational bandwidth with *γ*.

**Table S3. Dimensions and material properties of each member**

| Composition components | Parts | Parameters | | | Values |
| --- | --- | --- | --- | --- | --- |
| PRS | Rigid links | Geometric | *r* | | 4.4 mm |
|  |  |  | *L*_1_ | *δ* | 2.2mm |
|  |  |  | *L*_2_ |  |  |
|  |  |  | *L*_3_ |  |  |
|  |  |  | *L*_4_ |  |  |
|  |  |  | *L*_5_ |  |  |
|  |  |  | *L*_6_ |  |  |
|  |  |  | Thickness | | 0.3mm |
|  |  | Material | Modulus of elasticity | | 240Gpa |
|  |  |  | Density | | 1700kg/m^3^ |
|  | Flexible hinges | Geometric | *L_a_* | $\frac{\sqrt{2}}{2}\left(r-\delta\right)$ | 1.56 mm |
|  |  |  | *L_c_* |  |  |
|  |  |  | *L_d_* |  |  |
|  |  |  | *L_f_* |  |  |
|  |  |  | *L_b_* | $\frac{1}{2}\left(r-\delta\right)$ | 1.1mm |
|  |  |  | *L_e_* |  |  |
|  |  |  | Length | | 0.3mm |
|  |  |  | Thickness | | 0.007mm |
|  |  | Material | Modulus of elasticity | | 65 MPa |
|  |  |  | Density | | 1300kg/m^3^ |
| ARSs | Rigid links | Geometric | *l*_1_ | | 9.8mm |
|  |  |  | *l*_2_ | | 0.9mm |
|  |  |  | *l*_3_ | | 0.45mm |
|  |  |  | *l*_4_ | | 0.6mm |
|  |  |  | *l*_5_ | | 0.4mm |
|  |  |  | *l*_6_ | | 0.7mm |
|  |  |  | *l*_7_ | | 3mm |
|  |  |  | Width | | 4.4mm |
|  |  |  | Thickness | | 0.3mm |
|  |  | Material | Modulus of elasticity | | 240Gpa |
|  |  |  | Density | | 1700kg/m^3^ |
|  | Flexible hinges | Geometric | Length | | 0.3mm |
|  |  |  | Width | | 4.4 mm |
|  |  |  | Thickness | | 0.015mm |
|  |  | Material | Modulus of elasticity | | 65 MPa |
|  |  |  | Density | | 1300kg/m^3^ |
| Load | —— | | Mass | | 1g |
